# Influenza Infection, Newly Diagnosed Atrial Fibrillation, and One-Year Mortality in Hospitalized Adults: A Retrospective Cohort Study

**DOI:** 10.3390/jcm15218608

**Published:** 2026-11-05

**Authors:** Roman Sonkin, Anna Khalemsky, Carmi Bartal

**Affiliations:** 1 Internal Medicine Division, Soroka University Medical Center, Be’er-Sheva 8410101, Israel; carmiba@clalit.org.il; 2 Faculty of Health Sciences, Ben-Gurion University of the Negev, Be’er-Sheva 8410501, Israel; 3 Management Department, Jerusalem Multidisciplinary College, Jerusalem 9101001, Israel; anaha@jmc.ac.il

**Keywords:** atrial fibrillation, newly diagnosed atrial fibrillation, influenza, influenza vaccination, respiratory tract infections, mortality

## Abstract

**Background/Objectives**: Whether influenza increases the risk of newly diagnosed atrial fibrillation (NDAF) beyond the effect of acute illness is uncertain. We examined whether viral etiology and prior influenza vaccination (exposures) were associated with NDAF within one year (primary outcome), and whether NDAF was associated with one-year all-cause mortality (secondary outcome), in hospitalized adults. **Methods**: This retrospective cohort study included 21,653 adults hospitalized in Clalit Health Services facilities (2012–2020) who underwent respiratory-virus PCR testing: influenza A (*n* = 2829), influenza B (*n* = 992), other respiratory viruses (*n* = 561), and PCR-negative concurrent controls (*n* = 17,271). NDAF within 365 days was analyzed with cause-specific Cox and Fine–Gray models, including time windows; mortality with time-dependent Cox models; and vaccination with propensity score matching. **Results**: NDAF was recorded in 1228 patients (5.7%), 56% of them on the day of admission. Compared with PCR-negative patients, no viral etiology was associated with a higher hazard of NDAF (influenza A: HR 0.87, 95% CI 0.73–1.04; influenza B: HR 0.73, 95% CI 0.53–1.00), including within 30 days and after excluding day-0 events. NDAF was associated with higher one-year mortality (HR 1.68, 95% CI 1.52–1.87), especially when recorded after discharge (HR 3.04). Influenza vaccination was not associated with NDAF (HR 0.97, 95% CI 0.85–1.10). **Conclusions**: Among hospitalized adults tested for respiratory viruses, neither influenza nor prior vaccination was associated with NDAF compared with PCR-negative inpatients, who were not necessarily of similar acute illness severity. NDAF marked a higher hazard of one-year mortality.

## 1. Introduction

Atrial fibrillation (AF) is the most common sustained arrhythmia and a major burden on public health, society, and economies [1,2,3]. Established risk factors include age, male sex, physical inactivity, smoking, obesity, diabetes mellitus (DM), obstructive sleep apnea (OSA), hypertension, chronic heart failure (CHF), coronary artery disease (CAD), and chronic kidney disease (CKD) [4,5].

Patients with newly diagnosed AF (NDAF) face high early risks of death, stroke, systemic embolism, and major bleeding, especially within the first month after diagnosis [6]. Because acute physiological stressors often trigger AF, these triggers are a potential target for prevention; for example, seasonal influenza vaccination has been associated with a modestly lower risk of AF [7].

Inflammation and acute infection are closely linked to AF; inflammatory biomarkers predict the development of the arrhythmia [8,9], inflammation contributes to atrial structural remodeling [10,11,12,13], and AF is common in patients hospitalized with infection, including sepsis and COVID-19 [14,15,16,17,18,19].

Data on the association between influenza and AF are more limited. Influenza can affect the heart directly [20,21] and is a recognized trigger of myocardial infarction (MI) [22]. AF incidence was similar in patients hospitalized with COVID-19 or influenza and followed disease severity [23], and it may rise during the influenza season [24]. Influenza vaccination has been associated with lower all-cause mortality among patients with established AF [25] and with a lower risk of AF, although confounding by indication limits these observational findings [26,27].

It therefore remains unclear whether AF during respiratory viral infection reflects a virus-specific effect or the general effect of an acute illness severe enough to require hospitalization, acting on an underlying cardiovascular substrate. Most previous studies compared infected patients with uninfected community populations, a design that cannot separate these two explanations.

We therefore studied adults hospitalized with a respiratory-virus PCR test, using PCR-negative inpatients as concurrent controls. The primary aim was to determine whether influenza A or influenza B, compared with a negative PCR result, is associated with NDAF, after adjusting for demographic characteristics and cardiovascular and renal comorbidities. Secondary aims were to quantify the time-dependent association between NDAF and one-year all-cause mortality, to examine whether this association differs by viral etiology, and to examine the association between recent influenza vaccination and NDAF incidence. We use the term NDAF, as in earlier studies based on diagnosis codes [26], because the outcome was based on the first recorded diagnosis of AF, which may not coincide with the true onset of the arrhythmia.

## 2. Materials and Methods

### 2.1. Study Design

This retrospective cohort study used data from Clalit Health Services (Clalit), Israel’s largest health maintenance organization, which insures approximately half the national population, operates a nationwide hospital network, and maintains a centralized electronic health record (EHR) linking community and hospital care, laboratory results, medication dispensing, and vaccination data at the individual level. The study was conducted at Soroka University Medical Center, a Clalit-affiliated tertiary hospital, and data were extracted in August 2025 using the MDClone data platform (MDClone Ltd., Be’er Sheva, Israel). All adults hospitalized in Clalit facilities between 1 January 2012, and 29 February 2020, who underwent nasopharyngeal polymerase chain reaction (PCR) testing for respiratory viruses were eligible. The data extraction was limited to patients aged 18 years or older. Respiratory-virus testing was ordered at the discretion of the treating physicians, and the indication for testing was not recorded. The study period ended on 29 February 2020, shortly after the first COVID-19 case in Israel was confirmed (21 February 2020), to avoid the pandemic-related changes in testing, admission practices, and viral circulation.

### 2.2. Study Population

**Figure 1:**
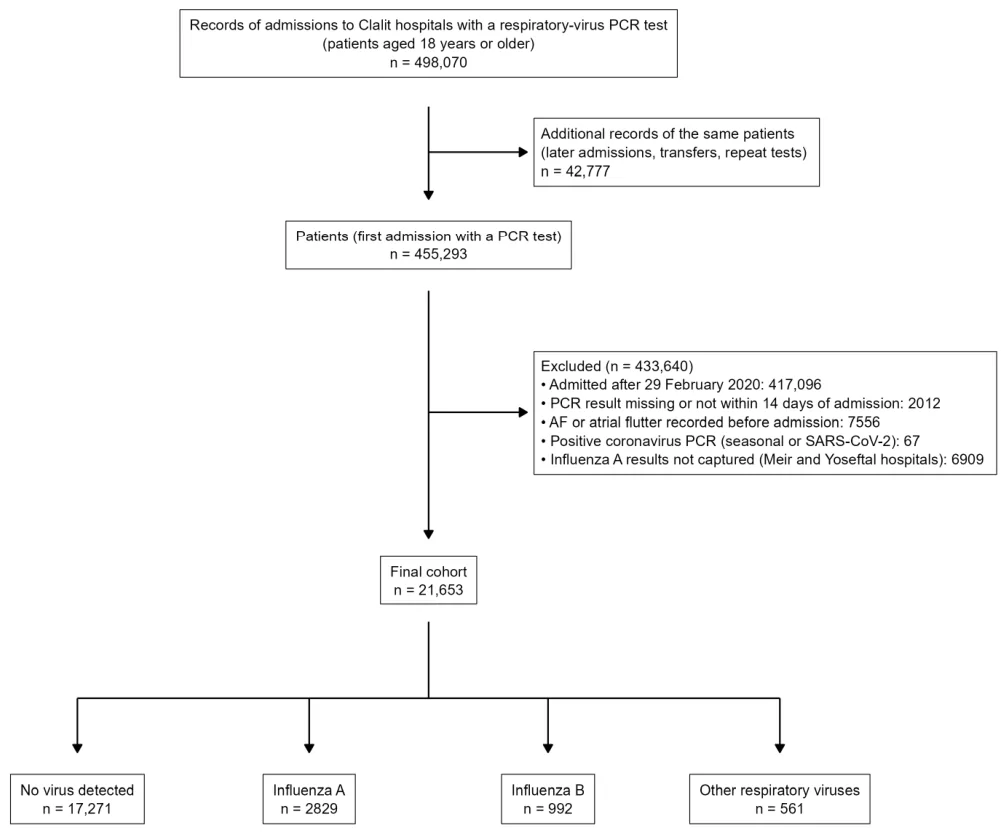
Flow diagram of cohort assembly.

Each patient contributed only the first admission with a respiratory-virus PCR result during the study period (the index admission); later admissions, department transfers, and repeat tests of the same patient were not used. The PCR result had to be available within 14 days of admission. Patients with any AF or atrial flutter documented before the index admission were excluded, using all prior records available in the Clalit EHR (no fixed lookback window was applied). The unified Clalit EHR (Ofek, based on Altera Digital Health dbMotion Solution) has linked community and hospital records since 2003 [28]; when it was established, all diagnoses already present in each patient’s medical record were entered into it with International Classification of Diseases, Ninth Revision (ICD-9) codes, so AF diagnosed before 2003 was also captured. Patients whose index PCR was positive for coronavirus were also excluded (*n* = 67; 7 with a test labelled SARS-CoV-2 and 60 with tests for other human coronaviruses), because coronaviruses were not part of the predefined exposure groups. Patients whose index admission was to Meir Medical Center (*n* = 6718) or Yoseftal Medical Center (*n* = 191) were also excluded, because the data extraction did not capture influenza A results from these hospitals: influenza A was recorded in only 24 of the 6718 patients tested at Meir (none from 2013 to 2018) and in none of the 191 patients tested at Yoseftal, whereas influenza B and other viruses were recorded at the usual rates. Results including these hospitals are reported as a sensitivity analysis. No patient was excluded for age or missing data. The number of patients at each step is shown in Figure 1.

### 2.3. Exposure Classification

Based on the index PCR result, patients were classified into four groups: influenza A, influenza B, other respiratory viruses (respiratory syncytial virus, parainfluenza virus, rhinovirus, human metapneumovirus, or adenovirus), and no virus detected (all panel targets negative). Results for each virus were extracted separately; when a patient had more than one result on the same date, a single category was assigned in a fixed order (influenza A, influenza B, other viruses, negative PCR), so each patient was assigned to one virological category. Viral co-detections were infrequent: 30 patients (0.14%) had positive results for more than one virus during the index admission (23 classified as influenza A and 7 as influenza B); the second virus was most often respiratory syncytial virus, and 22 patients (0.1%) classified as having no virus detected had a positive result later during the same admission.

For the mortality analysis, the main exposure was NDAF (defined in Section 2.4), entered as a time-dependent variable from the date it was first recorded.

Influenza vaccination was defined as documented receipt of at least one dose of influenza vaccine in the six months preceding the index admission. Immunization data are recorded centrally, capturing vaccinations administered across the organization’s community and hospital settings. Vaccination status was fixed at baseline; vaccinations given during follow-up were not considered.

### 2.4. Outcomes

The primary outcome was NDAF, defined as the first-ever addition of an AF diagnosis code to the patient’s EHR within 365 days of the index admission. Diagnoses in the Clalit EHR are coded with ICD-9 codes, and AF was identified by codes 427.3, 427.31, and 427.32. Records coded as atrial fibrillation, including combined atrial fibrillation and flutter, counted as NDAF, whereas records coded as atrial flutter alone (40 patients) were not counted. In Clalit, diagnoses are kept in a shared problem list that is updated by the treating physician at primary care visits and during daily hospital rounds; when AF is suspected, for example, because of an irregular pulse on examination, an electrocardiogram is obtained, and AF identified on the electrocardiogram is added to the problem list. The date of NDAF was the date AF was first entered into the EHR. In Israel, every hospitalized patient has an electrocardiogram at admission, and a new diagnosis of AF is entered only after the treating physician has reviewed the patient’s previous electrocardiograms and diagnoses. A single recorded diagnosis was sufficient; repeated codes were not required, electrocardiograms were not available for review in this study, and the coding definition was not validated in this study. Because the recorded date may differ from the true onset of the arrhythmia, we use the term newly diagnosed rather than new-onset AF. Time zero for all analyses was the date of the index admission. AF first recorded on the day of admission was counted as an NDAF event, because patients admitted with an acute illness are usually ill before arrival; a sensitivity analysis excluding these events is described in Section 2.6. Because follow-up started at admission, AF recorded later in the admission but before the respiratory sample was taken or its result was available was also counted. NDAF was further classified as in-hospital (first recorded during the index admission) or post-discharge. The secondary outcome was all-cause mortality within 365 days of the index admission, ascertained from the EHR.

### 2.5. Covariates

Baseline covariates were extracted from the EHR as of the index admission: age, sex, population sector (Jews, Arabs, Other), hypertension, CAD, CHF, DM, CKD, obesity, smoking status (never, former, current, unknown), and alcohol abuse. Comorbidities were defined by recorded diagnoses, and the absence of a diagnosis was coded as absent, so these variables had no missing values; age, sex, and population sector were complete. Smoking status was unknown for 2569 patients (11.9%). Because missing smoking status most likely reflected documentation practice rather than a clinical state, unknown status was kept as a separate category, and a complete-case sensitivity analysis was performed. The hospital of admission and the department that ordered the index PCR were recorded. A PCR ordered from an intensive care unit (ICU) was used as a marker of critical illness at the time of testing; cardiac ICUs were identified separately, because AF itself can lead to cardiac ICU admission, and only non-cardiac ICU testing was used for adjustment. Data on the reason for admission, indications for testing, laboratory values, and other measures of acute illness severity were not available.

### 2.6. Statistical Analysis

Continuous variables were summarized as mean ± standard deviation (SD) and categorical variables as counts (percentages). Differences in baseline characteristics were described with standardized mean differences (SMDs) rather than significance tests; Table 1 shows the largest absolute SMD between any virus group and patients with no virus detected, and SMDs below 0.1 were regarded as negligible. The crude one-year incidence of NDAF across viral etiologies was compared with Fisher’s exact test, using simulated *p*-values based on 10,000 replicates.

Because death precludes the subsequent occurrence of AF, the cumulative incidence of NDAF was estimated with the Aalen–Johansen estimator, treating death as a competing event. Unadjusted survival according to NDAF status was described with the Simon–Makuch method [29], in which patients contribute person-time to the no-NDAF group until NDAF is recorded and to the NDAF group thereafter, which avoids immortal-time (guarantee-time) bias.

The association between viral etiology and NDAF was assessed with a multivariable cause-specific Cox proportional hazards model (censoring at death), with the PCR-negative group as reference, adjusted for age, sex, population sector, hypertension, CAD, CHF, DM, CKD, obesity, smoking status, and alcohol abuse. As a sensitivity analysis accounting for the competing risk of death, a Fine–Gray subdistribution hazards model (sHR) with the same covariates was fitted; its estimates are reported as sHRs. Covariates were chosen in advance as established risk factors for AF and death that were available in the EHR; no data-driven variable selection was used, and age was entered as a continuous linear term. The numbers of events are reported with all hazard ratios (HRs).

To examine whether an acute effect of infection was diluted over one year of follow-up, the NDAF model was repeated within time windows after admission. Follow-up was split at days 30 and 90, and an interaction between viral etiology and time window (days 0–30, 31–90, and 91–365) gave a window-specific HR for each etiology. In a separate model, follow-up was split at the date of discharge, to estimate HRs for the in-hospital and post-discharge periods.

The association between NDAF and all-cause mortality was assessed with a multivariable Cox model in which NDAF was entered as a time-dependent covariate, such that patients contributed unexposed person-time until NDAF was first recorded and exposed person-time thereafter, which avoids immortal-time bias. The model was adjusted for viral etiology and all covariates above. Effect modification by viral etiology was tested by adding NDAF × etiology interaction terms. Because the interaction coefficients alone do not give the association within each etiology, the HR for NDAF within each viral etiology was derived as a linear combination of the main-effect and interaction coefficients, with 95% CIs from the model variance–covariance matrix; the numbers of NDAF events and of deaths after NDAF are reported for each stratum. The interaction analyses were regarded as exploratory, and Holm-adjusted *p*-values for the three interaction terms are reported. To examine whether the prognostic significance of NDAF depended on the timing of onset, in-hospital and post-discharge NDAF were entered as two separate time-dependent indicators in a single adjusted model.

The association between influenza vaccination and NDAF was evaluated with an unadjusted Cox model, a multivariable Cox model in the full cohort, and a propensity score–matched analysis. The target estimand was the average effect of vaccination in vaccinated patients (average treatment effect in the treated) within this population of hospitalized, PCR-tested adults. Because vaccination precedes infection and may influence whether a patient acquires influenza, viral etiology is a potential mediator rather than a confounder of this association. In the primary analysis, viral etiology was therefore not included in the propensity score model or in the outcome models; a specification that included it is reported as a sensitivity analysis. The propensity score was estimated by logistic regression of vaccination status on age, sex, population sector, comorbidities, smoking status, and alcohol abuse. Vaccinated and unvaccinated patients were matched 1:1 by nearest-neighbor matching without replacement using a caliper of 0.05 standard deviations of the propensity score; this narrow caliper was chosen to favor close matches over sample size. Vaccinated patients without a match within the caliper were excluded, so the estimate applies to vaccinated patients who had comparable unvaccinated counterparts. Balance was assessed with Standardized Mean Differences (SMDs; values < 0.1 were considered acceptable) and by inspecting propensity score distributions before and after matching. The outcome model in the matched sample retained covariate adjustment (doubly robust estimation), and robust standard errors were clustered on matched pairs. Sensitivity analyses (i) added year of admission, and then year and season, to the propensity score model (season matters here because vaccination was defined by a dose in the preceding six months), (ii) excluded patients vaccinated fewer than 14 days before admission, when a protective immune response is not yet expected, and (iii) included viral etiology in both models. A subgroup analysis was performed among patients hospitalized with influenza A or B; because this analysis conditions on infection and hospitalization after vaccination, it is prone to selection (collider) bias and was regarded as exploratory. Owing to sparse cells in this subgroup, population sector was collapsed to two categories (Jews, non-Jews).

Additional sensitivity analyses of the NDAF model (i) excluded NDAF events recorded on the day of admission (and, separately, on days 0–1), in both the cause-specific and the Fine–Gray models, to address AF that may have been present before, or may have contributed to, the admission; (ii) added calendar year of admission, and then calendar year and season (winter, spring, summer, autumn), as covariates, to account for changes in testing and viral circulation over time and for seasonal differences in the reasons for admission; (iii) were restricted to admissions in December–March, the main influenza season, when most influenza-positive patients were admitted; (iv) used patients with other respiratory viruses, all of whom had a confirmed viral infection, as the reference group; (v) were restricted to patients with known smoking status (complete-case analysis); (vi) were restricted to patients whose PCR result was available within 5 days of admission; and (vii) adjusted for, or excluded, patients whose PCR was ordered from an ICU. A further sensitivity analysis repeated the main NDAF model in the original cohort, including Meir and Yoseftal Medical Centers.

The proportional-hazards assumption was evaluated using scaled Schoenfeld residuals; global and covariate-specific tests are reported in Table S11. The NDAF model included 17 parameters for 1228 events (about 72 events per parameter), and the mortality interaction model included 21 parameters for 4085 deaths (about 195 events per parameter). All tests were two-sided with α = 0.05. Analyses were performed in R version 4.2.3 (R Foundation for Statistical Computing, Vienna, Austria), using the survival (version 3.5-7), MatchIt (version 4.5.4), cmprsk (version 2.2-11), cobalt (version 4.5.1), gtsummary (version 1.7.2), and survminer (version 0.4.9) packages.

### 2.7. Ethics

The study was approved by the Institutional Review Board of Soroka University Medical Center (approval number 0176-24-SOR, approval date 24 July 2024), with a waiver of informed consent given the retrospective design and use of de-identified data.

### 2.8. Generative Artificial Intelligence

The authors used generative AI tools to convert a pre-specified statistical analysis plan into R code, to perform iterative data-consistency checks, to assist with language editing, and to provide a critical review of the analytic methods and outputs, whose suggestions were evaluated and either accepted or rejected by the authors. Decisions on study design, statistical methods, and interpretation were made by the authors. All code was reviewed and run by the authors (A.K. and R.S.), who verified all outputs and take full responsibility for the analyses and their interpretation.

## 3. Results

### 3.1. Baseline Patient Characteristics

Overall, 21,653 adults met the inclusion criteria (Figure 1): 17,271 (79.8%) with no virus detected, 2829 (13.1%) with influenza A, 992 (4.6%) with influenza B, and 561 (2.6%) with other respiratory viruses (Table 1). In total, 11,302 patients (52%) were female, and the mean age was 62 ± 20 years. Patients with other respiratory viruses were the oldest (67 ± 18 years) and carried the greatest comorbidity burden (hypertension 67%, CHF 18%, CKD 8.7%, obesity 50%), whereas the influenza groups were slightly younger (influenza A, 61 ± 21; influenza B, 60 ± 22) and somewhat less comorbid than PCR-negative patients (hypertension 53% and 51% vs 57%). Patients who developed NDAF during follow-up were older (76 ± 12 vs. 62 ± 21 years) and had more hypertension (82% vs. 55%), CAD (39% vs. 23%), CHF (22% vs. 12%), and obesity (51% vs. 39%) than those who did not (Table S7). Overall, 9209 patients (42.5%) had been vaccinated against influenza within 6 months before admission. Vaccinated patients were markedly older (70 ± 17 vs. 57 ± 21 years) and more comorbid (hypertension 72% vs. 45%; CAD 33% vs. 18%; DM 38% vs. 20%) than unvaccinated patients; their characteristics before and after propensity score matching are shown in Table S8.

### 3.2. Newly Diagnosed Atrial Fibrillation by Viral Etiology

**Figure 2:**
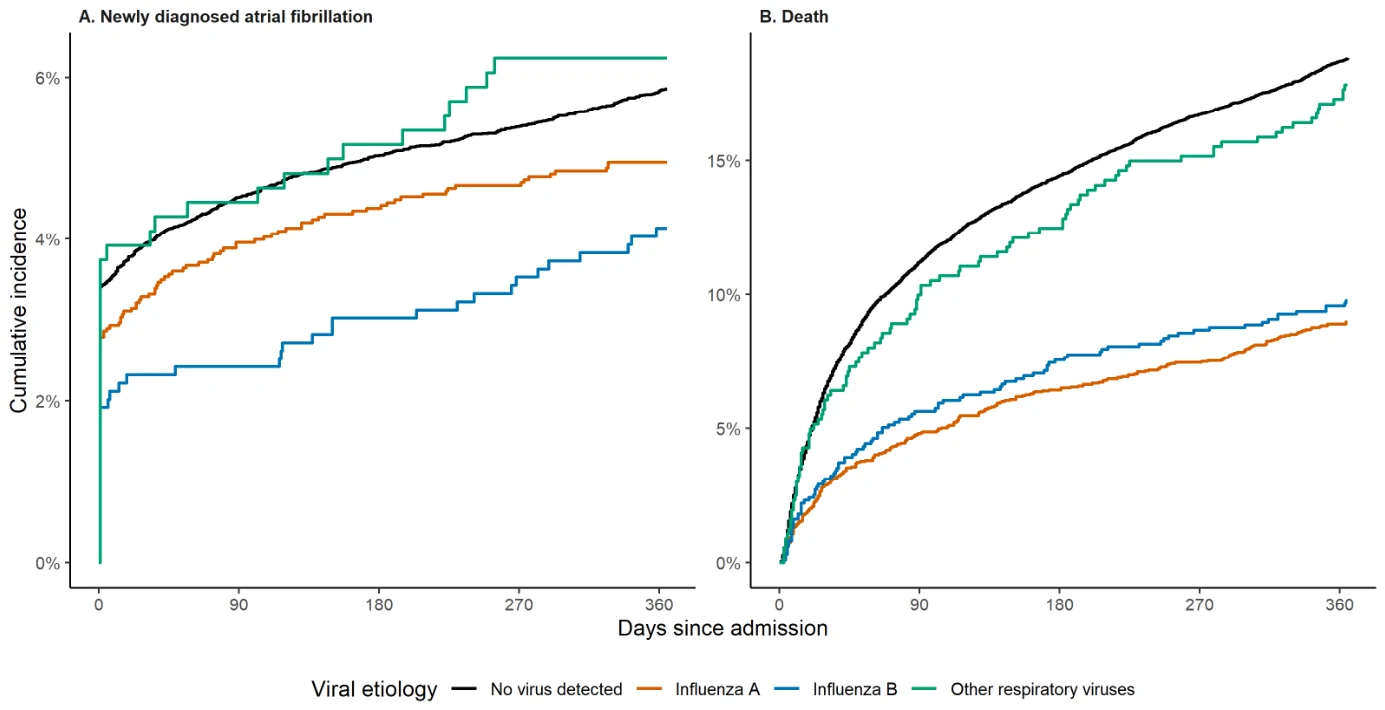
Cumulative incidence of NDAF (**A**) and of death (**B**) by viral etiology during the 365 days after admission. Aalen–Johansen estimates, with death treated as a competing event for NDAF. The step at day 0 reflects NDAF recorded on the day of admission.

Within 365 days of admission, NDAF was recorded in 1228 patients (5.7%). The one-year cumulative incidence differed modestly across groups (5.9% in PCR-negative patients, 4.9% in influenza A, 4.1% in influenza B, and 6.2% in other respiratory viruses; *p* = 0.032; Table S1), with the lowest crude incidence observed in the influenza groups. Cumulative incidence curves accounting for the competing risk of death showed the same ordering (Figure 2). In every group, a substantial proportion of events was first documented during the index admission (732 [59.6%] in-hospital vs. 496 [40.4%] post-discharge; Table S10).

After multivariable adjustment, no viral etiology was associated with a higher hazard of NDAF relative to PCR-negative patients: influenza A HR 0.87 (95% CI 0.73–1.04; *p* = 0.118), influenza B HR 0.73 (0.53–1.00; *p* = 0.047), and other respiratory viruses HR 0.90 (0.64–1.26; *p* = 0.524) (Table S2); NDAF events: 1012 in PCR-negative patients, 140 in influenza A, 41 in influenza B, and 35 in other respiratory viruses. The lower hazard ratio in influenza B was of borderline significance and disappeared when NDAF recorded on the day of admission was excluded (see below). The Fine–Gray competing-risk analysis was concordant (influenza A sHR 0.91, 0.76–1.08; influenza B sHR 0.76, 0.56–1.04; other viruses sHR 0.91, 0.65–1.28; Table S5). Other factors associated with NDAF were older age (HR 1.04 per year), male sex (1.14), hypertension (1.41), CHF (1.17), and obesity (1.38).

In the time-window analysis (Table S10), the HRs for influenza A and influenza B, compared with PCR-negative patients, were 0.89 (95% CI 0.72–1.11) and 0.63 (0.42–0.96) in days 0–30 (821 events), 1.16 (0.71–1.91) and 0.17 (0.02–1.22) in days 31–90 (121 events), and 0.69 (0.46–1.02) and 1.21 (0.74–1.98) in days 91–365 (286 events). During the index admission, the HRs were 0.90 (0.71–1.13) and 0.66 (0.42–1.01), and after discharge 0.83 (0.63–1.10) and 0.83 (0.53–1.30). No window showed a higher hazard of NDAF with influenza. The hazard was lower for influenza B in days 0–30; the estimates for days 31–90 and 91–365 rested on few events (for example, one event in influenza B in days 31–90) and did not follow a consistent pattern.

Results were similar in the sensitivity analyses (Table S13). After exclusion of NDAF recorded on the day of admission (685 events), the HRs for influenza A and influenza B were 0.88 (0.68–1.14) and 0.89 (0.59–1.36) in the cause-specific model and 0.97 (0.75–1.26) and 0.99 (0.65–1.49) in the Fine–Gray model, so the lower hazard in influenza B was confined to NDAF recorded on the day of admission; after adjustment for calendar year, 0.87 (0.73–1.04) and 0.74 (0.54–1.01), and for calendar year and season, 0.84 (0.71–1.01) and 0.72 (0.52–0.99); among admissions in December–March (16,169 patients), 0.86 (0.71–1.03) and 0.75 (0.54–1.03); with other respiratory viruses as the reference group, 0.97 (0.67–1.41) and 0.81 (0.52–1.28); and in the complete-case analysis (19,084 patients), 0.88 (0.73–1.08) and 0.68 (0.48–0.98). Estimates were also similar when the analysis was limited to patients tested within 5 days of admission, adjusted for a PCR ordered from a non-cardiac ICU, or limited to patients not tested in an ICU (HRs 0.89–0.90 for influenza A and 0.75–0.77 for influenza B). In the original cohort including Meir and Yoseftal Medical Centers (28,562 patients), the HRs were 0.89 (0.75–1.05) and 0.77 (0.59–1.00).

### 3.3. Newly Diagnosed Atrial Fibrillation and One-Year Mortality

**Figure 3:**
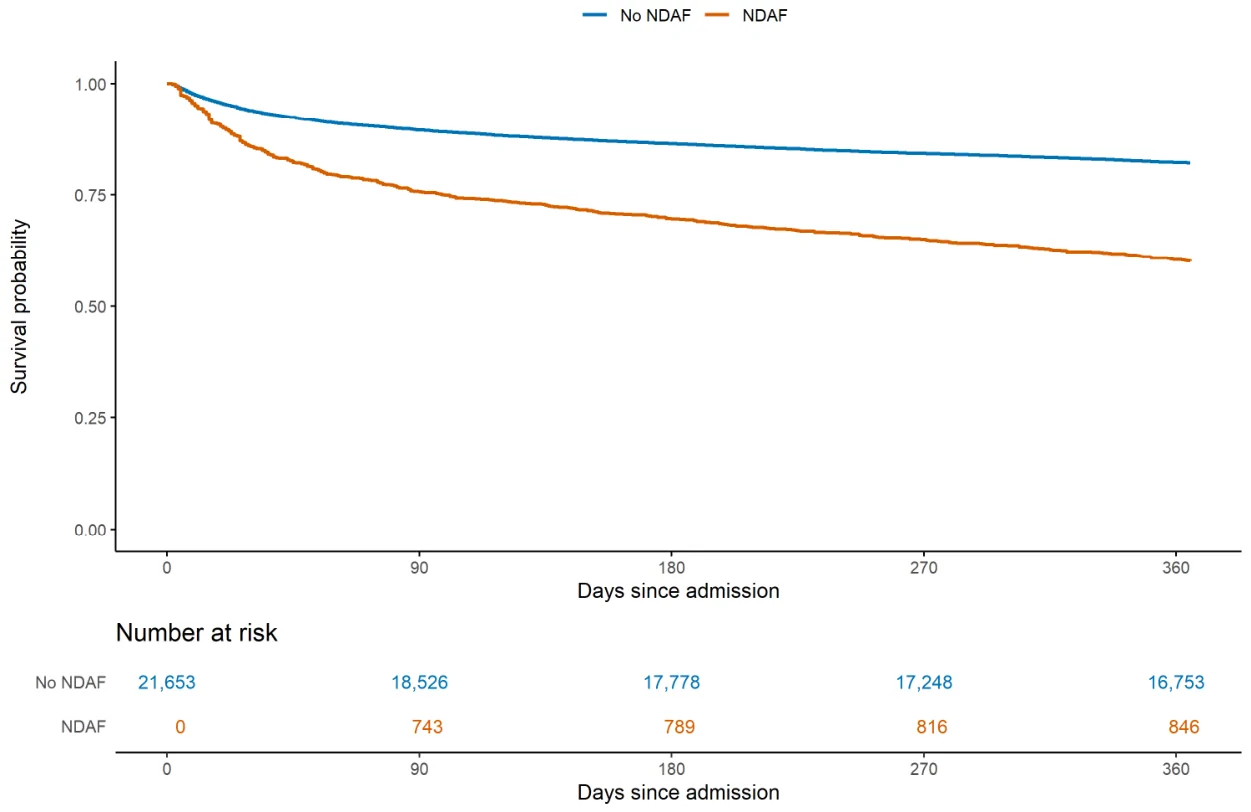
Simon–Makuch estimates of one-year survival according to NDAF status. Patients contribute person-time to the no-NDAF group until NDAF is first recorded and to the NDAF group thereafter; the numbers at risk are shown below the curves. Because patients join the NDAF group only when NDAF is recorded, its number at risk starts at zero and rises over time. The adjusted association, with NDAF modelled as a time-dependent covariate, is shown in Table 2.

In total, 4085 patients (18.9%) died within 365 days. Crude mortality was substantially higher among patients who developed NDAF than among those who did not (31% vs. 18%; Table S1), although this crude comparison does not account for when NDAF occurred. In Simon–Makuch curves, in which patients contribute person-time to the NDAF group only after NDAF is recorded, estimated one-year survival was 60.1% (95% CI 57.1–63.3) with NDAF and 82.1% (81.6–82.7) without NDAF (Figure 3). In the multivariable time-dependent Cox model, NDAF was associated with all-cause mortality (HR 1.68, 95% CI 1.52–1.87; 384 deaths after NDAF; *p* < 0.001; Table 2). Additional adjustment for a PCR ordered from a non-cardiac ICU gave a similar estimate (HR 1.61, 95% CI 1.45–1.79). Relative to PCR-negative patients, all virus-positive groups had lower adjusted mortality: influenza A HR 0.47 (0.42–0.53), influenza B HR 0.53 (0.43–0.64), and other respiratory viruses HR 0.81 (0.67–0.98). These inverse associations most likely reflect differences in the reasons for admission between the groups rather than a protective effect of viral infection (see Discussion). Other covariates associated with mortality included age (HR 1.04 per year), male sex (1.24), CHF (1.38), DM (1.28), CKD (1.30), current smoking (1.11), and alcohol abuse (1.49), whereas obesity (0.82) and CAD (0.92) showed inverse associations.

When NDAF was split by timing, both in-hospital NDAF (HR 1.32, 95% CI 1.16–1.51; 236 deaths after NDAF) and post-discharge NDAF (HR 3.04, 2.57–3.59; 148 deaths after NDAF) were associated with mortality (both *p* < 0.001; Table S6). This association was therefore not confined to events recorded during the index illness and was strongest for NDAF first recorded after discharge.

In the exploratory interaction model (Table S3), the association between NDAF and mortality did not differ significantly by viral etiology (interaction HR 1.82, 95% CI 0.96–3.42, *p* = 0.065, Holm-adjusted *p* = 0.196, for influenza B; 1.15, 0.76–1.75, *p* = 0.508, for influenza A; and 0.81, 0.39–1.68, *p* = 0.570, for other respiratory viruses). Derived from this model, the HR for NDAF was 1.66 (95% CI 1.48–1.85) in PCR-negative patients (1012 NDAF events; 339 deaths after NDAF), 1.91 (1.27–2.86) in influenza A (140; 26), 3.01 (1.61–5.61) in influenza B (41; 11), and 1.34 (0.65–2.75) in other respiratory viruses (35; 8) (Table S12). The higher estimate in influenza B rests on 11 deaths after NDAF and should be regarded as hypothesis-generating.

The proportional-hazards assumption was met for viral etiology in the NDAF model (*p* = 0.41). In the mortality model, the assumption was met for NDAF (*p* = 0.18); when NDAF was split by timing, in-hospital NDAF showed a borderline departure (*p* = 0.049) and post-discharge NDAF did not (*p* = 0.28); for vaccination in the matched model, *p* = 0.95. Global tests were significant, as expected in a cohort of this size, reflecting departures in baseline covariates (Table S11).

### 3.4. Influenza Vaccination and Newly Diagnosed Atrial Fibrillation

Because vaccinated patients were markedly older and more comorbid than unvaccinated patients (Section 3.1; Table S8), crude NDAF incidence was higher among vaccinated patients (7.3% vs. 4.5%; *p* < 0.001; Figure S1), and the unadjusted hazard ratio suggested apparent harm (HR 1.68, 95% CI 1.50–1.88; *p* < 0.001). This association was fully attenuated after multivariable adjustment in the whole cohort, both without adjustment for viral etiology (HR 0.99, 95% CI 0.88–1.12) and with it (HR 1.00, 0.89–1.12; *p* = 0.935; Table S4).

In the primary propensity score model, which did not include viral etiology, matching yielded 7414 pairs (14,828 patients); 1795 vaccinated patients (19.5%) had no match within the caliper and were excluded. After matching, all matching covariates, including age and the propensity score itself, had SMDs below 0.05 (viral etiology, which was not a matching variable, had an SMD of 0.08), and the propensity score distributions were nearly superimposed (Table S8; Figures S3 and S4). NDAF occurred in 485 vaccinated and 490 unvaccinated matched patients (Figure S2). In the doubly robust matched analysis, with standard errors clustered on matched pairs, vaccination was not associated with NDAF (HR 0.97, 95% CI 0.85–1.10; *p* = 0.591; Table 3). The results were similar when viral etiology was included in both models (HR 1.04, 95% CI 0.92–1.18), after calendar year was added to the propensity score model (HR 0.99, 95% CI 0.87–1.13), after calendar year and season were added (6551 pairs; HR 0.98, 95% CI 0.86–1.13), and after exclusion of the 398 patients vaccinated fewer than 14 days before admission (HR 0.99, 95% CI 0.88–1.13; Table S13).

In the exploratory subgroup of patients hospitalized with influenza A or B (*n* = 3821; 1595 [42%] vaccinated; 181 NDAF events; about 13 events per model parameter), the adjusted hazard ratio for vaccination was 1.35 (95% CI 0.98–1.85; *p* = 0.063; Table 4). Because this estimate conditions on infection and hospitalization after vaccination, it is prone to collider bias; its interpretation is addressed in the Discussion.

## 4. Discussion

In this cohort of 21,653 adults hospitalized in a health organization that covers about half of the Israeli population, we report four main findings. First, compared with PCR-negative inpatients, influenza infection was not associated with a higher hazard of NDAF within one year or within the first 30 days after admission. Second, NDAF was associated with a 68% higher hazard of one-year all-cause mortality, whether it was first recorded in-hospital or post-discharge; this is a prognostic association and does not show that AF itself causes death. Third, in an exploratory analysis, this association did not differ significantly by viral etiology. Fourth, prior influenza vaccination was not associated with NDAF in this hospitalized, PCR-tested population. To our knowledge, this was the first study to directly compare influenza A and influenza B with respect to NDAF.

We compared patients with influenza with PCR-negative inpatients, who were also ill enough to be admitted and tested. This control group is heterogeneous: it probably includes patients with bacterial pneumonia, sepsis, exacerbations of chronic cardiopulmonary disease, and other acute illnesses that can themselves trigger atrial arrhythmia; in a population-based study, new AF was almost seven times more likely in patients with severe sepsis than in other hospitalized adults, particularly with a respiratory source of infection or respiratory failure [30]. Previous studies mostly compared infected patients with community-dwelling populations or used within-person designs, and found that acute respiratory infection transiently increases the risk of cardiovascular events such as MI and stroke [31,32]. For AF specifically, a nationwide case–control study from Taiwan, which compared patients with newly diagnosed AF with age- and sex-matched controls from the general population and identified influenza by diagnosis codes and antiviral treatment, found that influenza infection in the preceding year was associated with an 18% higher risk of AF (odds ratio 1.18) and vaccination with a 12% lower risk (odds ratio 0.88) [26]; in an Israeli cohort, SARS-CoV-2 infection was associated with a higher risk of NDAF within 30 days [19]. In contrast, among patients hospitalized with COVID-19 or influenza, AF incidence was similar and was associated with inflammation and illness severity [23], which is closer to our findings. Our design answers a different question. Our results show no excess NDAF risk with influenza relative to other acute illnesses that led to hospitalization and testing, beyond the measured baseline characteristics; they do not show that influenza has no arrhythmogenic effect compared with remaining well, and they are therefore compatible with these earlier studies. Our confidence intervals exclude an excess of the size reported in Taiwan relative to other hospitalized patients (upper limits 1.04 for influenza A and 1.00 for influenza B), consistent with the view that much of the excess seen against community controls may reflect acute illness in general. Influenza B was associated with a slightly lower hazard of NDAF (HR 0.73), but this difference disappeared when AF recorded on the day of admission was excluded (HR 0.89), which suggests that it reflects the larger share of PCR-negative admissions in which AF was part of the presenting illness rather than a protective effect of influenza B. In our cohort, the one-year cumulative incidence of NDAF was 4.1–6.2% in every group, and the factors most strongly associated with NDAF were age, male sex, hypertension, CHF, and obesity [4,5]. Three of these factors, hypertension, CHF, and obesity, drive atrial cardiomyopathy: structural, electrical, and functional remodeling of the atria that contributes to both the development and the perpetuation of AF [11]. Through atrial fibrosis, inflammation, and oxidative stress, this remodeling creates a pro-arrhythmic and pro-thrombotic atrial substrate, and atrial fibrosis can progress in patients with heart failure who do not have AF [11]. One interpretation consistent with our data is that acute illness, viral or not, acts as a trigger in patients who already have such a substrate, so that the substrate rather than the pathogen largely determines who develops AF. Atrial structure and function were not measured in our cohort, so this interpretation remains a plausible framework rather than a finding of our study.

NDAF was associated with a higher one-year mortality. Modeled as a time-dependent covariate to avoid immortal-time bias, NDAF was associated with a 68% higher hazard of one-year mortality (HR 1.68). New AF has likewise been associated with higher mortality in patients with sepsis, although with smaller effect sizes: the adjusted relative risk of in-hospital death was 1.07 [30], and among Medicare sepsis survivors the five-year hazard of death was modestly higher (HR 1.04), with higher risks of heart failure and ischemic stroke; AF recurred after discharge in more than half of these patients [16]. Crucially, this risk was not confined to the index admission: NDAF first recorded after discharge was associated with a more than three-fold higher mortality hazard (HR 3.04). Post-discharge NDAF is often recorded during a new contact with health care, such as a readmission, which may itself signal clinical deterioration. These estimates describe prognosis, not causation. NDAF may be a marker of worsening illness, such as decompensated heart failure, ischemia, or other complications that were not measured and that increase both the chance that AF is detected, because sicker patients are monitored more closely, and the risk of death; adjustment for baseline comorbidities cannot separate the effect of AF from that of time-varying disease severity. In the sepsis cohort, adjustment for acute organ dysfunction reduced the mortality association of new AF from a relative risk of 1.33 to 1.07 [30]; because we could adjust for acute illness only through ICU testing, part of the association we observed may likewise reflect illness severity. With this caveat, NDAF after an acute respiratory illness may identify patients with an underlying atrial and cardiovascular substrate [11] who could benefit from structured cardiovascular follow-up, a hypothesis that should be tested prospectively. Our cohort also reproduced the established obesity paradox [33,34]; obesity was associated with a higher hazard of NDAF (HR 1.38) but lower hazard of mortality (HR 0.82).

In an exploratory interaction analysis, the association between NDAF and mortality appeared stronger in patients with influenza B (HR 3.01, 95% CI 1.61–5.61) than in PCR-negative patients (HR 1.66), but the difference was not statistically significant (interaction *p* = 0.065; Holm-adjusted *p* = 0.196). This estimate rests on 41 NDAF events and 11 deaths after NDAF in the influenza B group and may be a chance finding. Previous studies found similar outcomes among hospitalized patients with influenza A and influenza B after adjustment for baseline characteristics, as well as comparable short-term mortality [35,36], although influenza B has been associated with myocardial involvement in some case series [37]. We therefore regard this result as hypothesis-generating; it should not be used to define a high-risk subgroup unless it is replicated in an independent cohort.

Prior observational studies have suggested that influenza vaccination may lower the risk of AF [7,26,27]. In our data, vaccinated patients exhibited a higher crude incidence of NDAF (7.3% vs. 4.5%), which reflected their older age and greater burden of established cardiovascular disease. After propensity score matching, prior vaccination was not associated with NDAF (HR 0.97, 95% CI 0.85–1.10). The protective cardiovascular benefits of the influenza vaccine have been shown most clearly for MI [38]; our data do not show a similar association for NDAF, but this result must be read within its scope. It applies to hospitalized adults who were tested for respiratory viruses, and it does not show that influenza vaccination offers no protection against AF in the general population. Vaccination may lower the chance of acquiring influenza, of being hospitalized, and of being tested, and therefore of entering our cohort; these selection mechanisms cannot be resolved within a hospitalized cohort. Matching balances measured covariates only, and observational vaccination studies are prone to healthy-user effects and to differences in health care use that are not captured by diagnosis codes [39]. Vaccination status was also measured only at baseline.

The apparent signal in the subgroup hospitalized with influenza A or B warrants explicit caution: this estimate conditions on breakthrough infection severe enough to cause hospitalization despite vaccination, a collider structure [40] in which vaccinated patients who are nonetheless hospitalized with influenza are selected for frailty, immune senescence, or poor vaccine response. Combined with the modest number of events relative to model parameters (about 13 events per parameter), sparse cells within covariate strata, borderline significance, and the multiplicity of comparisons across the study, this subgroup estimate should not be interpreted as evidence of vaccine-attributable arrhythmic risk; the matched full-cohort estimate is the primary result.

### Strengths and Limitations

This study has several strengths: a large cohort drawn from a health organization with hospitals across Israel and a centralized EHR that links community and hospital care; a comparator group of concurrently hospitalized, PCR-tested patients; time-dependent and competing-risk survival models; and propensity score matching for the vaccination analysis. It also has important limitations. First, NDAF was defined by the first recorded diagnosis in the EHR rather than continuous telemetry or adjudicated electrocardiograms. The coding definition was not validated in this study, and a single recorded diagnosis was sufficient. In a US health plan, ICD-9 codes for AF had a sensitivity of 95% and a specificity of 99%, but on chart review 23% of patients with a first AF code had earlier AF [41]. Subclinical or paroxysmal AF, AF seen on electrocardiograms or telemetry but never coded, and AF in patients who had no contact with health care during follow-up may have been missed, and the electrocardiogram tracings were not available for review in this study. AF diagnosed before the patient joined Clalit, or outside the organization, may have been recorded for the first time during follow-up and counted as new; and the recorded date may follow the true onset. AF recorded on the day of admission (56% of events) may have been present before, or may have contributed to, the admission; excluding these events did not change the results. Detection may also differ between groups: patients with longer hospital stays, more monitoring, more readmissions, or more outpatient visits have more opportunities for AF to be found, so identical coding rules do not guarantee equal ascertainment.

Second, residual confounding is possible. The PCR-negative group is heterogeneous, and we had no data on the reason for admission, laboratory values, or most measures of acute illness severity (for example, sepsis, hypoxemia, mechanical ventilation, vasopressor use, or acute kidney injury); ICU care was captured only through the department that ordered the PCR, and adjustment for it did not change the results. The lower adjusted mortality of virus-positive patients almost certainly reflects these differences in case mix rather than a protective effect of viral infection, and it shows how different the comparison groups were. Our estimates should therefore be interpreted as associations beyond the measured baseline characteristics.

Third, the cohort was selected by the requirement for respiratory PCR testing, which was done at the discretion of the treating physicians; testing practices varied over time (the annual number of tested patients rose more than tenfold between 2012 and 2019), between hospitals, and with clinical presentation and severity. Adjustment for calendar year and season and restriction to the influenza season did not materially change the results, but the cohort does not represent all hospitalized adults or all patients admitted with respiratory illness [40].

Fourth, the vaccination analysis has specific limitations. Vaccination status was measured only at baseline. Matching balanced measured characteristics only, so healthy-user effects and differences in health care use may remain. Because the cohort included only hospitalized, tested patients, the vaccination results apply to hospitalized, tested adults and do not show the effect of vaccination on AF in the general population.

Fifth, 30 patients with more than one virus were classified by a fixed order, and 22 patients in the no-virus group had a positive result later in the same admission; these patients (0.2% of the cohort) are too few to affect the estimates materially. The “other respiratory viruses” group combines several pathogens.

Sixth, the influenza B interaction and the vaccination subgroup analysis were based on few events and involved multiple comparisons, and they should be regarded as exploratory.

Seventh, smoking status was unknown in 11.9% of patients.

Finally, the study ended before the COVID-19 pandemic. Respiratory-virus testing, vaccination practices, influenza circulation, admission thresholds, and the management of acute respiratory illness have changed since 2020, and our findings may not apply directly to the current setting. Clalit insures about half of the Israeli population, almost all patients were hospitalized in Clalit hospitals, and results may differ in other health systems. In addition, influenza A results from two hospitals were not captured by the data extraction; these hospitals were excluded, and including them gave similar results (Table S13).

Future studies should include measures of acute illness severity, such as the reason for admission, vital signs, laboratory values, and intensive care use, so that influenza can be compared with other acute illnesses of similar severity. The effect of influenza vaccination on AF should be studied in the whole population, including patients who are treated in the community and never hospitalized, ideally with designs that reduce healthy-user bias, such as self-controlled case series or target-trial emulation, or within randomized vaccine trials.

## 5. Conclusions

Among hospitalized adults tested for respiratory viruses, neither influenza infection nor prior influenza vaccination was associated with the one-year hazard of NDAF when compared with PCR-negative hospitalized patients, who were not necessarily of equivalent acute illness severity. Instead, the risk of NDAF was associated primarily with age and with cardiovascular and metabolic comorbidities. When NDAF was recorded, whether during the index admission or after discharge, it was associated with a 68% higher hazard of one-year mortality. NDAF in this setting should therefore not be dismissed as a benign, transient event; it marks patients at higher risk, and whether structured cardiovascular follow-up improves their outcomes should be evaluated in prospective studies.

## Figures and Tables

**Table 1:** Baseline characteristics of the study population by viral etiology.

Characteristic	Total, N = 21,653 ^1^	No Virus Detected, N = 17,271 ^1^	Influenza A, N = 2829 ^1^	Influenza B, N = 992 ^1^	Other Respiratory Viruses, N = 561 ^1^	SMD ^2^
**Age, years**	62 ± 20	63 ± 20	61 ± 21	60 ± 22	67 ± 18	0.21
**Sex**						0.12
Female	11,302 (52%)	8895 (52%)	1516 (54%)	572 (58%)	319 (57%)	
Male	10,351 (48%)	8376 (48%)	1313 (46%)	420 (42%)	242 (43%)	
**Population sector**						0.05
Jews	15,357 (71%)	12,228 (71%)	2015 (71%)	722 (73%)	392 (70%)	
Arabs	4552 (21%)	3616 (21%)	619 (22%)	199 (20%)	118 (21%)	
Other	1744 (8.1%)	1427 (8.3%)	195 (6.9%)	71 (7.2%)	51 (9.1%)	
**Hypertension**	12,226 (56%)	9836 (57%)	1506 (53%)	506 (51%)	378 (67%)	0.22
**Coronary artery disease**	5195 (24%)	4196 (24%)	631 (22%)	207 (21%)	161 (29%)	0.10
**Congestive heart failure**	2663 (12%)	2193 (13%)	282 (10.0%)	85 (8.6%)	103 (18%)	0.16
**Diabetes mellitus**	6041 (28%)	4871 (28%)	727 (26%)	248 (25%)	195 (35%)	0.14
**Chronic kidney disease**	851 (3.9%)	700 (4.1%)	71 (2.5%)	31 (3.1%)	49 (8.7%)	0.19
**Obesity**	8497 (39%)	6819 (39%)	1071 (38%)	329 (33%)	278 (50%)	0.20
**Smoking status**						0.14
Never	11,585 (54%)	9101 (53%)	1579 (56%)	584 (59%)	321 (57%)	
Former	3462 (16%)	2802 (16%)	422 (15%)	150 (15%)	88 (16%)	
Current	4037 (19%)	3303 (19%)	498 (18%)	149 (15%)	87 (16%)	
Unknown	2569 (12%)	2065 (12%)	330 (12%)	109 (11%)	65 (12%)	
**Alcohol abuse**	597 (2.8%)	515 (3.0%)	50 (1.8%)	21 (2.1%)	11 (2.0%)	0.08
**Influenza vaccination within 6 months**	9209 (43%)	7302 (42%)	1189 (42%)	406 (41%)	312 (56%)	0.27
**PCR ordered from an intensive care unit**	1120 (5.2%)	995 (5.8%)	87 (3.1%)	21 (2.1%)	17 (3.0%)	0.19

^1^ *n* (%); mean ± SD. ^2^ Largest absolute standardized mean difference between any virus group and patients with no virus detected; values below 0.1 indicate a negligible difference. Baseline characteristics by NDAF status, and of vaccinated and unvaccinated patients before and after propensity score matching, are shown in Tables S7 and S8.

**Table 2:** Multivariable time-dependent Cox proportional hazards regression for one-year all-cause mortality.

Characteristic ^1^	HR	95% CI	*p*-Value
NDAF (time-dependent)	1.68	1.52–1.87	<0.001
Viral etiology			
No virus detected	—	—	
Influenza A	0.47	0.42–0.53	<0.001
Influenza B	0.53	0.43–0.64	<0.001
Other respiratory viruses	0.81	0.67–0.98	0.034
Age, years	1.04	1.04–1.04	<0.001
Sex			
Female	—	—	
Male	1.24	1.16–1.33	<0.001
Population sector			
Jews	—	—	
Arabs	0.87	0.79–0.95	0.003
Other	1.02	0.92–1.13	0.672
Hypertension	0.96	0.88–1.04	0.313
Coronary artery disease	0.92	0.85–0.99	0.020
Congestive heart failure	1.38	1.27–1.49	<0.001
Diabetes mellitus	1.28	1.20–1.37	<0.001
Chronic kidney disease	1.30	1.13–1.49	<0.001
Obesity	0.82	0.76–0.87	<0.001
Smoking status			
Never	—	—	
Former	1.07	0.98–1.17	0.122
Current	1.11	1.02–1.22	0.022
Unknown	0.99	0.90–1.08	0.780
Alcohol abuse	1.49	1.27–1.75	<0.001

^1^ *n* = 21,653; 4085 deaths within one year. NDAF (1228 patients) was entered as a time-dependent covariate. HR, hazard ratio; CI, confidence interval.

**Table 3:** Propensity score–matched Cox model for influenza vaccination and NDAF.

Characteristic ^1^	HR	95% CI	*p*-Value
Influenza vaccination within 6 months	0.97	0.85–1.10	0.591
Age, years	1.04	1.04–1.05	<0.001
Sex			
Female	—	—	
Male	1.13	1.00–1.29	0.058
Population sector			
Jews	—	—	
Arabs	0.85	0.70–1.04	0.120
Other	0.90	0.73–1.11	0.340
Hypertension	1.34	1.11–1.62	0.002
Coronary artery disease	1.09	0.94–1.26	0.266
Congestive heart failure	1.17	0.98–1.39	0.075
Diabetes mellitus	1.02	0.89–1.17	0.746
Chronic kidney disease	1.09	0.82–1.47	0.543
Obesity	1.34	1.17–1.52	<0.001
Smoking status			
Never	—	—	
Former	0.99	0.83–1.19	0.926
Current	1.20	1.00–1.45	0.055
Unknown	1.03	0.87–1.23	0.702
Alcohol abuse	0.74	0.46–1.19	0.212

^1^ Matched cohort: 7414 pairs (14,828 patients; 975 NDAF events). Adjusted for the covariates shown; viral etiology was not included. Robust standard errors clustered on matched pairs. HR, hazard ratio; CI, confidence interval.

**Table 4:** Exploratory subgroup analysis of influenza vaccination and NDAF in patients hospitalized with influenza A or B.

Characteristic ^1^	HR	95% CI	*p*-Value
Influenza vaccination within 6 months	1.35	0.98–1.85	0.063
Age, years	1.05	1.03–1.06	<0.001
Sex			
Female	—	—	
Male	1.02	0.75–1.40	0.898
Population sector			
Jews	—	—	
Non-Jews	1.00	0.71–1.43	0.981
Hypertension	1.01	0.67–1.52	0.951
Coronary artery disease	1.37	0.98–1.92	0.064
Congestive heart failure	1.11	0.74–1.67	0.615
Diabetes mellitus	0.94	0.68–1.30	0.717
Chronic kidney disease	1.35	0.66–2.79	0.411
Obesity	1.48	1.09–2.01	0.012
Smoking status			
Never	—	—	
Former	1.16	0.76–1.78	0.483
Current	1.54	1.00–2.38	0.050
Unknown	1.12	0.75–1.68	0.579
Alcohol abuse	0.33	0.05–2.40	0.276

^1^ *n* = 3821; 181 NDAF events (about 13 events per model parameter). Population sector was collapsed to two categories because of sparse cells. HR, hazard ratio; CI, confidence interval.

## Data Availability

The data presented in this study are not publicly available due to privacy restrictions of Clalit Health Services.

## Supplementary Materials

The following supporting information can be downloaded at: https://www.mdpi.com/article/10.3390/jcm15218608/s1. Figure S1: Cumulative incidence of NDAF (A) and of death (B) by influenza vaccination status in the full (unmatched) cohort: vaccinated (*n* = 9209) and unvaccinated (*n* = 12,444) patients. Aalen–Johansen estimates, with death treated as a competing event. Figure S2: Cumulative incidence of NDAF (A) and of death (B) by influenza vaccination status in the propensity score–matched cohort (7414 matched pairs; 14,828 patients). Aalen–Johansen estimates, with death treated as a competing event. Figure S3: Covariate balance before and after propensity score matching. Absolute standardized mean differences (SMDs) for each covariate and for the propensity score before and after matching. The dashed vertical line marks the 0.1 threshold for acceptable balance. Figure S4: Propensity score distributions before and after matching. Kernel density estimates of the propensity score for vaccinated and unvaccinated patients before and after matching. Table S1: Crude one-year incidence of NDAF by viral etiology and crude one-year mortality by NDAF status. Table S2: Multivariable cause-specific Cox regression for NDAF by viral etiology. Table S3: Time-dependent Cox model with an NDAF × viral etiology interaction for one-year mortality. Table S4: Cox models for influenza vaccination and NDAF in the full (unmatched) cohort. Table S5: Fine–Gray competing-risk regression for NDAF, with death as the competing event. Table S6: Time-dependent Cox model with separate indicators for in-hospital and post-discharge NDAF and one-year mortality. Table S7: Baseline characteristics by NDAF status. Table S8: Baseline characteristics of vaccinated and unvaccinated patients before and after propensity score matching, with standardized mean differences. Table S9: Number of tested patients, viral etiology, and influenza vaccination by calendar year, 2012–2020. Table S10: NDAF by viral etiology within time windows after admission: numbers at risk, events, and hazard ratios. Table S11: Proportional-hazards assumption tests (scaled Schoenfeld residuals) for the main models. Table S12: Association between NDAF and one-year mortality within each viral etiology, derived from the interaction model, with numbers of patients, NDAF events, and deaths. Table S13: Sensitivity analyses for NDAF by viral etiology (Part A) and for influenza vaccination and NDAF (Part B).

## Abbreviations

The following abbreviations are used in this manuscript:

AF	Atrial Fibrillation
CAD	Coronary Artery Disease
CHF	Congestive Heart Failure
CI	Confidence Interval
CKD	Chronic Kidney Disease
COVID-19	Coronavirus Disease 2019
DM	Diabetes Mellitus
EHR	Electronic Health Record
HR	Hazard Ratio
ICD	International Classification of Diseases
MI	Myocardial Infarction
NDAF	Newly Diagnosed Atrial Fibrillation
OSA	Obstructive Sleep Apnea
PCR	Polymerase Chain Reaction
SD	Standard Deviation
sHR	subdistribution Hazard Ratio
SMD	Standardized Mean Difference
