# Supplementary material for: Influenza Infection, Newly Diagnosed Atrial Fibrillation, and One-Year Mortality in Hospitalized Adults: A Retrospective Cohort Study
Source: J Clin Med. 2026 Nov 5;15(21):8608. doi: 10.3390/jcm15218608 (PMC13763677; doi:10.3390/jcm15218608)

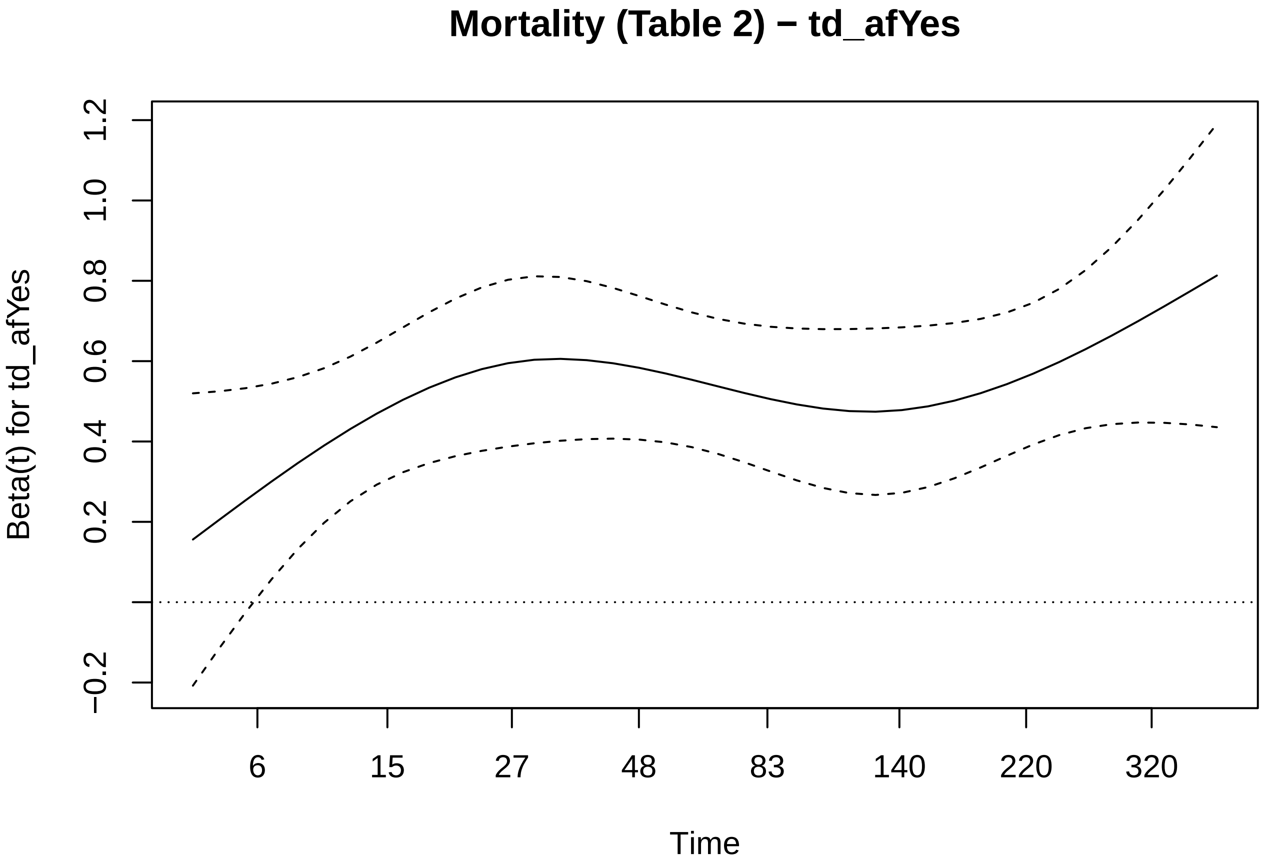

Mortality (Table 2) − td_afYes
Beta(t) for td_afYes
−0.2
0.2
0.4
0.6
0.8
1.0
1.2
6
15
27
48
83
140
220
320
Time

Mortality (Table 2) − groupInfluenza A

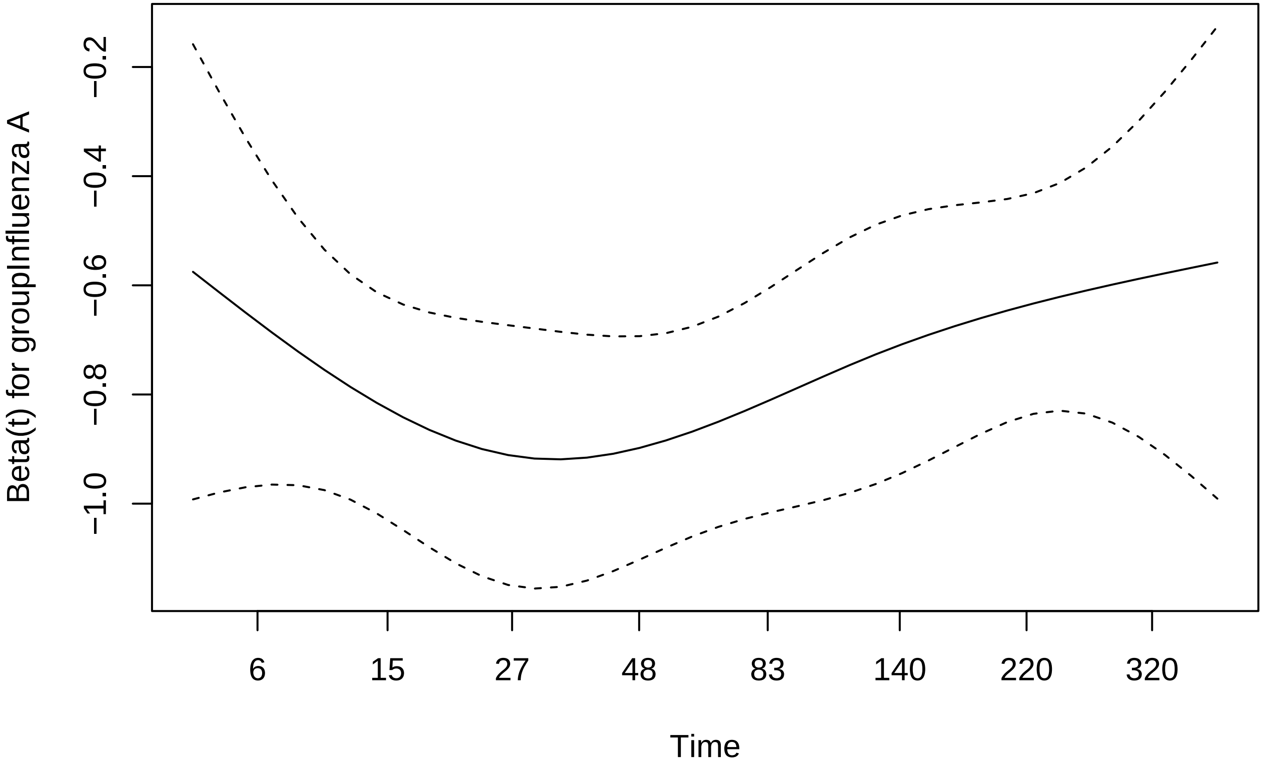

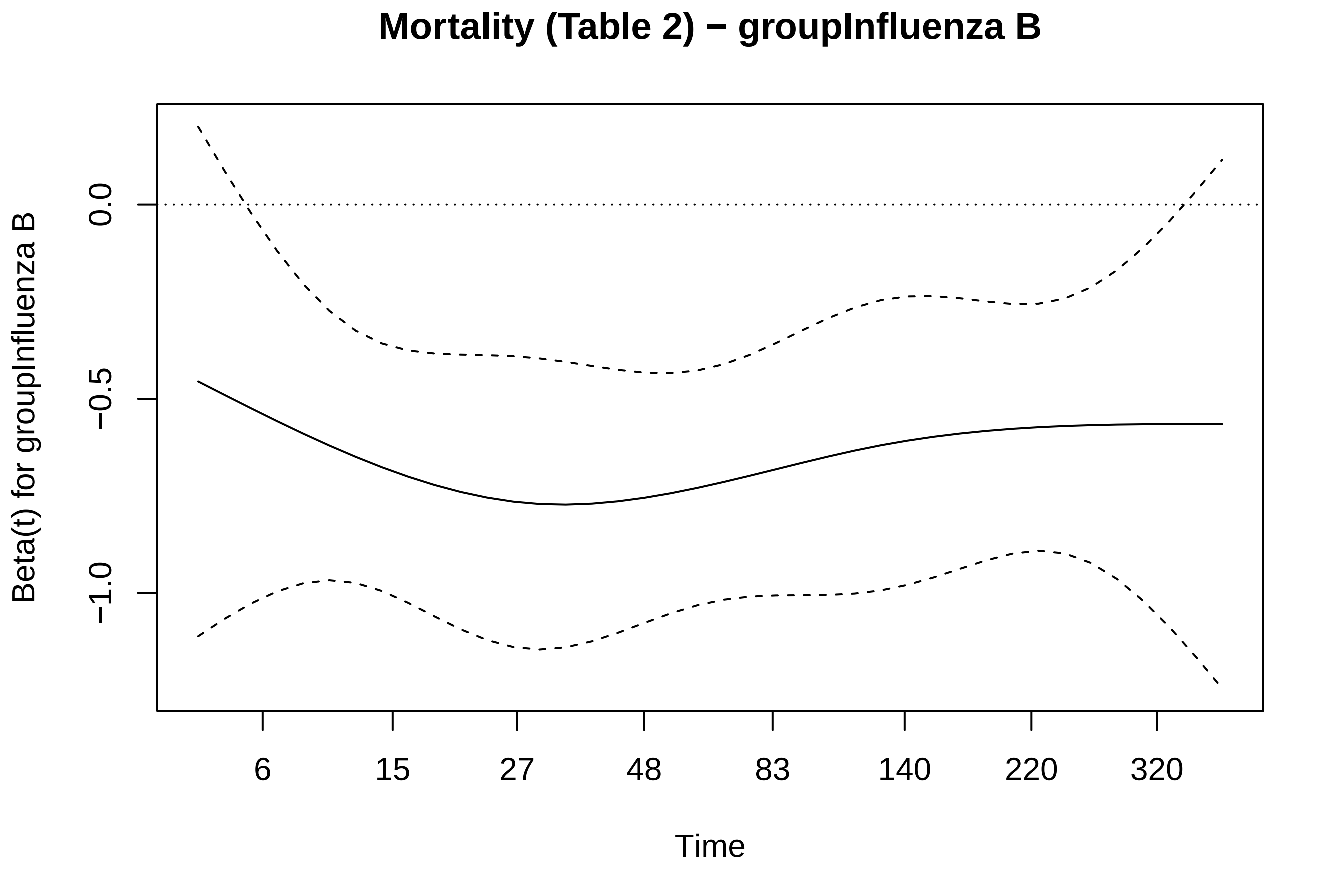
Mortality (Table 2) − groupInfluenza B
Beta(t) for groupInfluenza B
0.0
−0.5
−1.0
6
15
27
48
83
140
220
320
Time

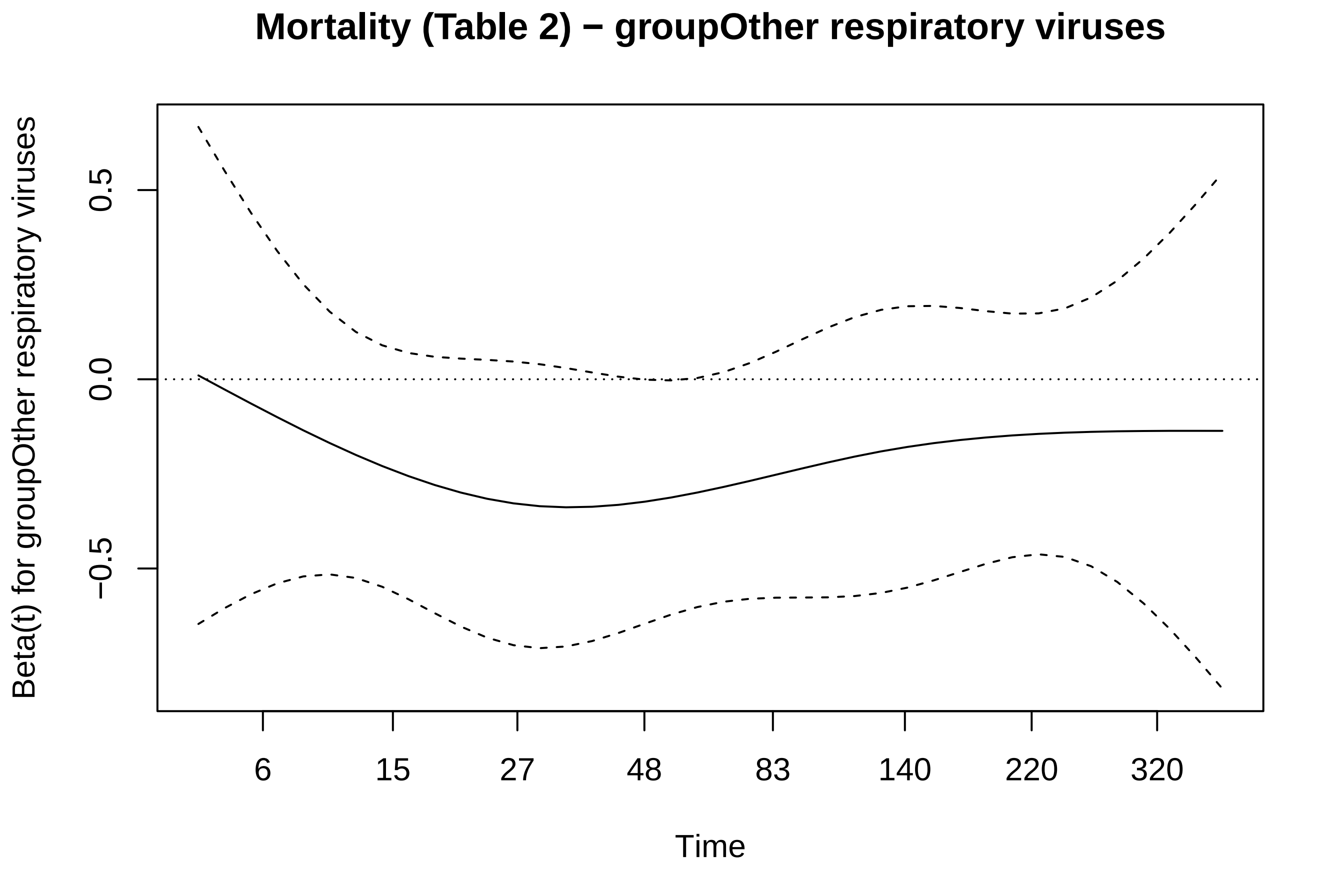

Mortality (Table 2) − groupOther respiratory viruses
Beta(t) for groupOther respiratory viruses
0.5
0.0
−0.5
6
15
27
48
83
140
220
320
Time

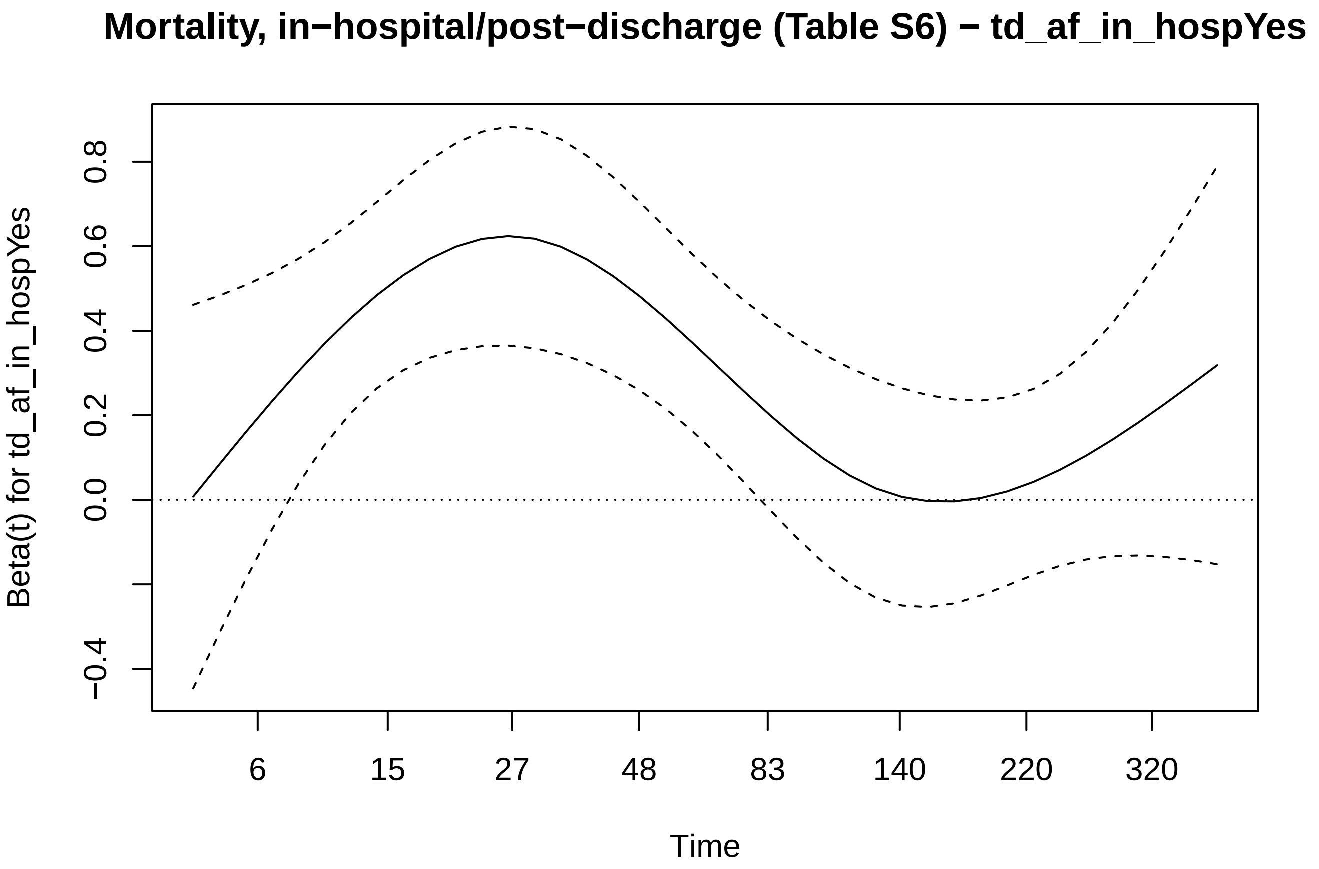

Mortality, in−hospital/post−discharge (Table S6) − td_af_in_hospYes
Beta(t) for td_af_in_hospYes
−0.4
0.0
0.2
0.4
0.6
0.8
6
15
27
48
83
140
220
320
Time

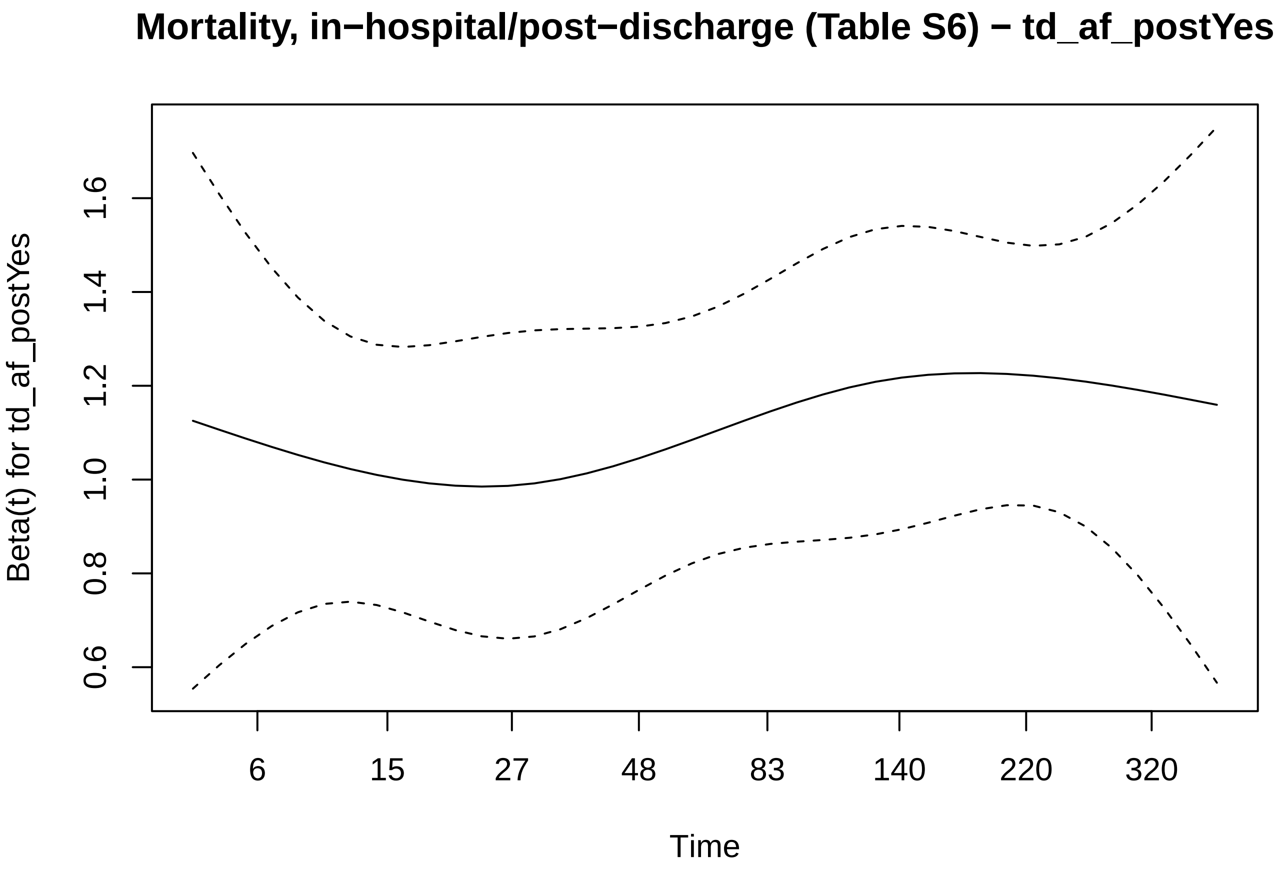
Mortality, in−hospital/post−discharge (Table S6) − td_af_postYes
Beta(t) for td_af_postYes
0.6
0.8
1.0
1.2
1.4
1.6
6
15
27
48
83
140
220
320
Time

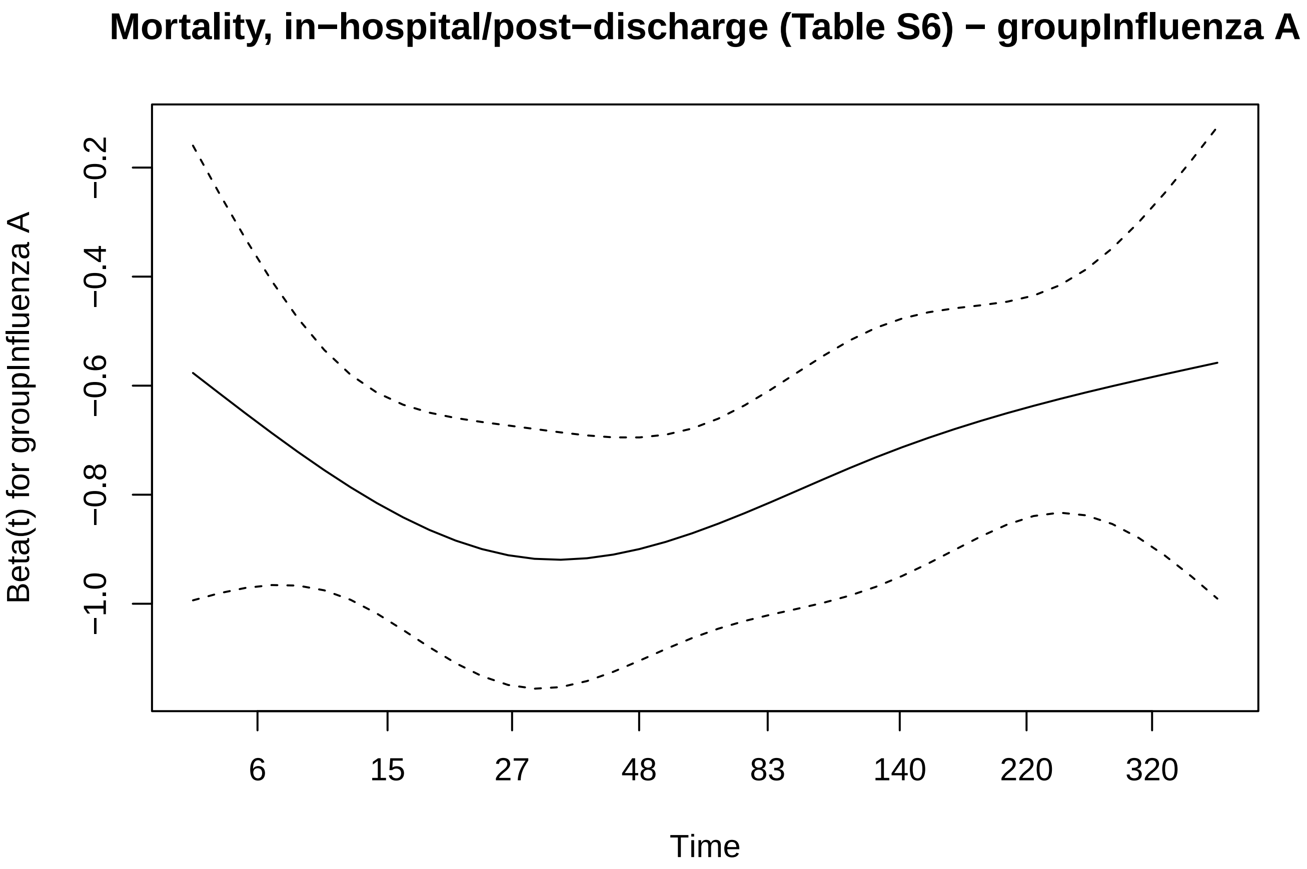
Mortality, in−hospital/post−discharge (Table S6) − groupInfluenza A
Beta(t) for groupInfluenza A
−0.2
−0.4
−0.6
−0.8
−1.0
6
15
27
48
83
140
220
320
Time

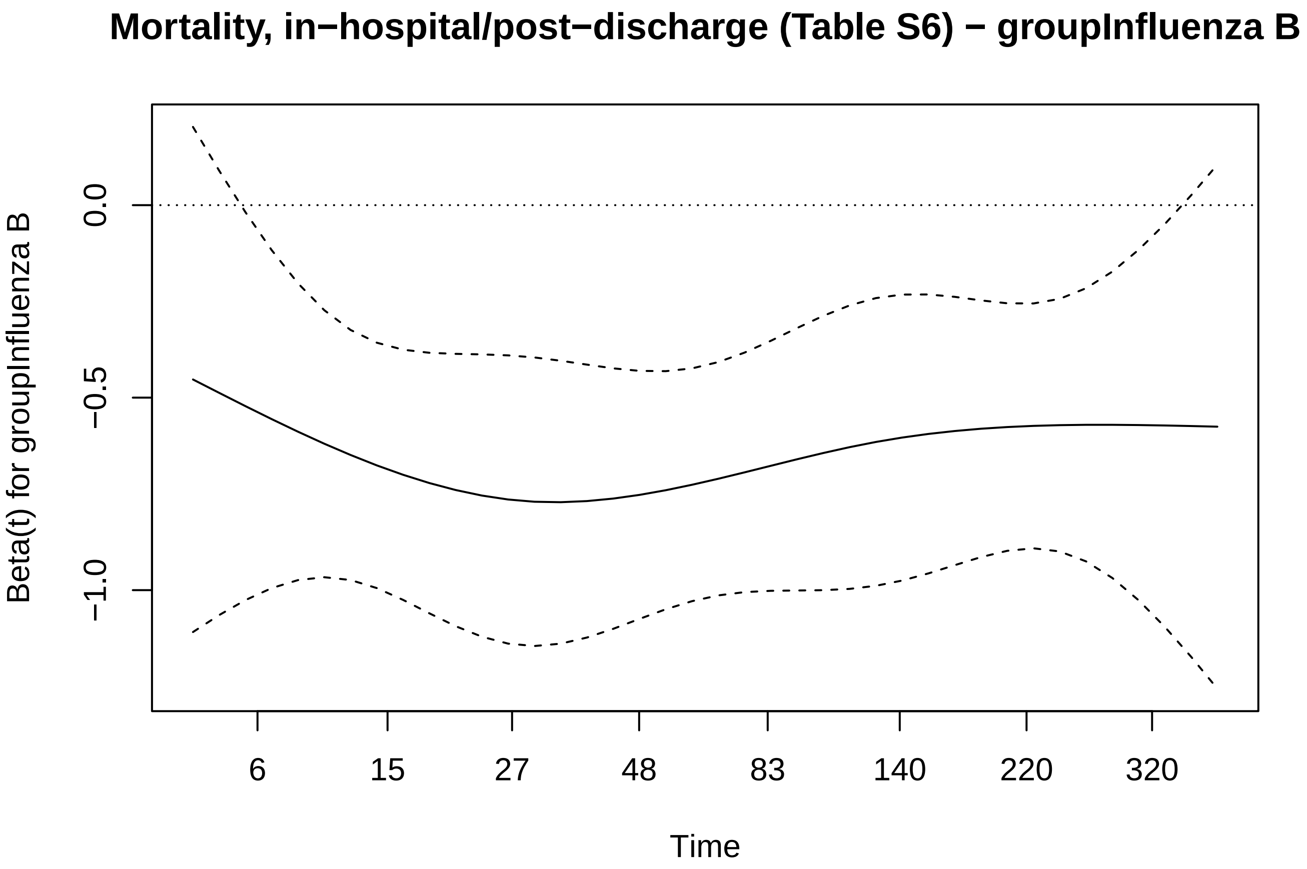

Mortality, in−hospital/post−discharge (Table S6) − groupInfluenza B
Beta(t) for groupInfluenza B
0.0
−0.5
−1.0
6
15
27
48
83
140
220
320
Time

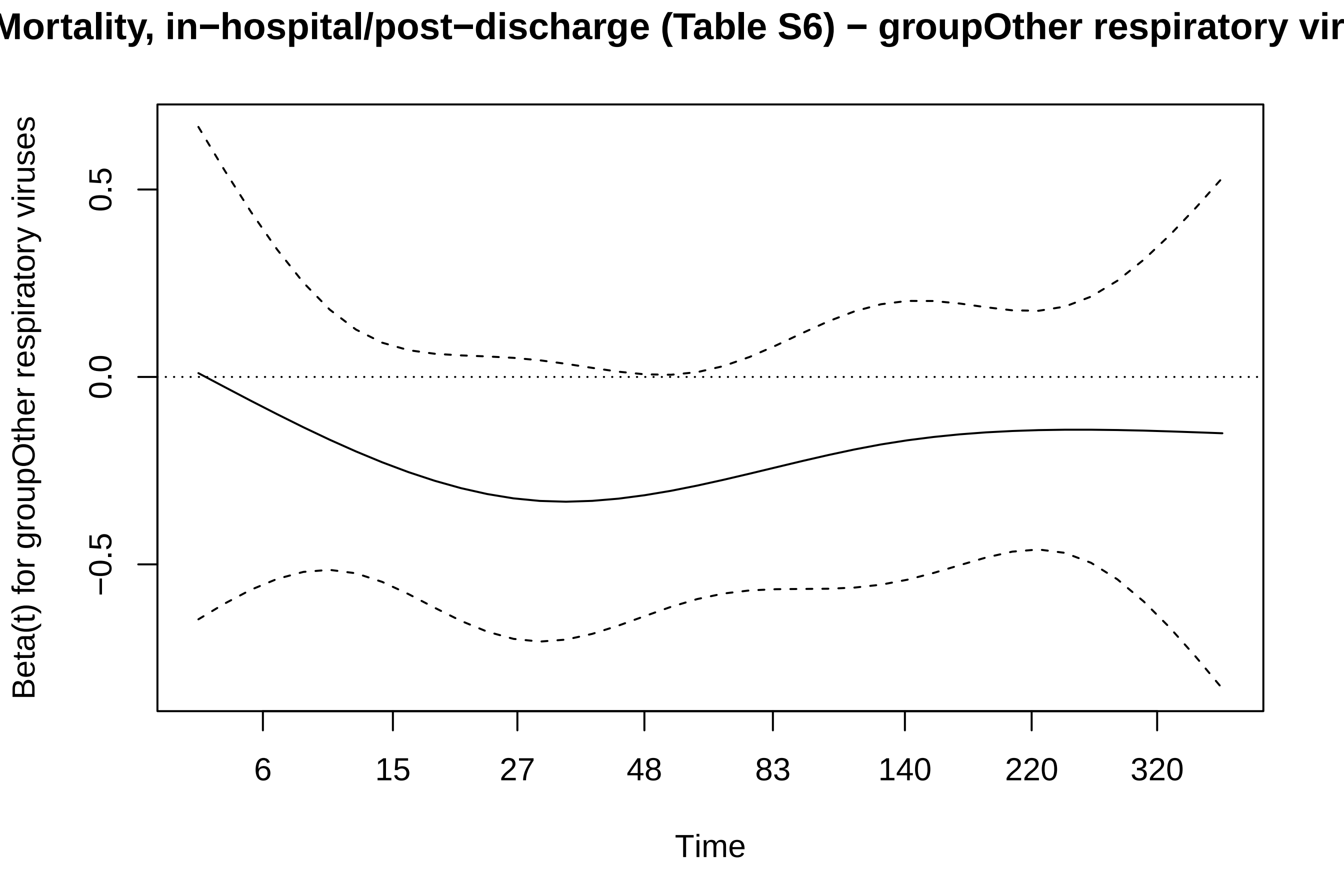
Mortality, in−hospital/post−discharge (Table S6) − groupOther respiratory vir
Beta(t) for groupOther respiratory viruses
0.5
0.0
−0.5
6
15
27
48
83
140
220
320
Time

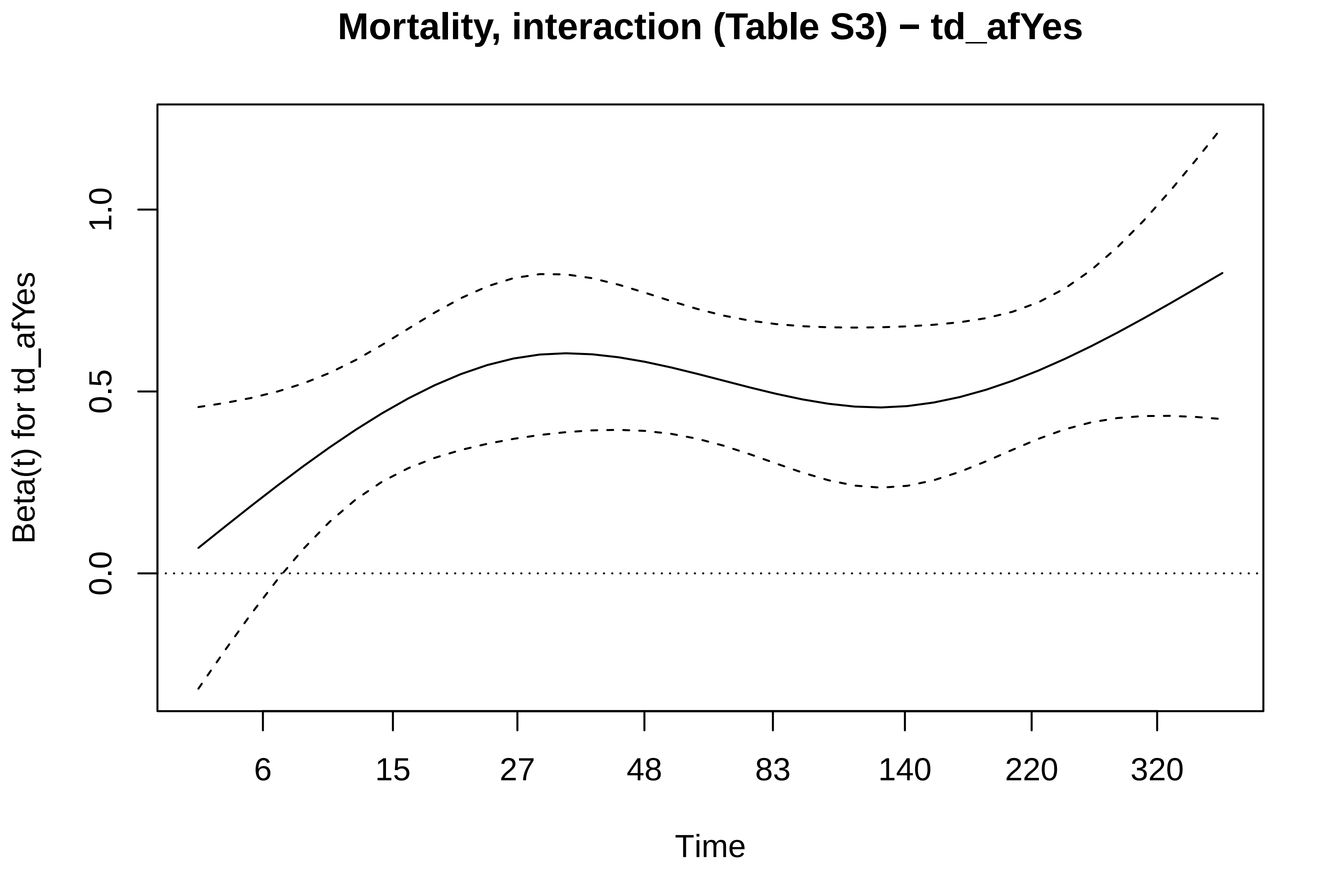
Mortality, interaction (Table S3) − td_afYes
Beta(t) for td_afYes
1.0
0.5
0.0
6
15
27
48
83
140
220
320
Time

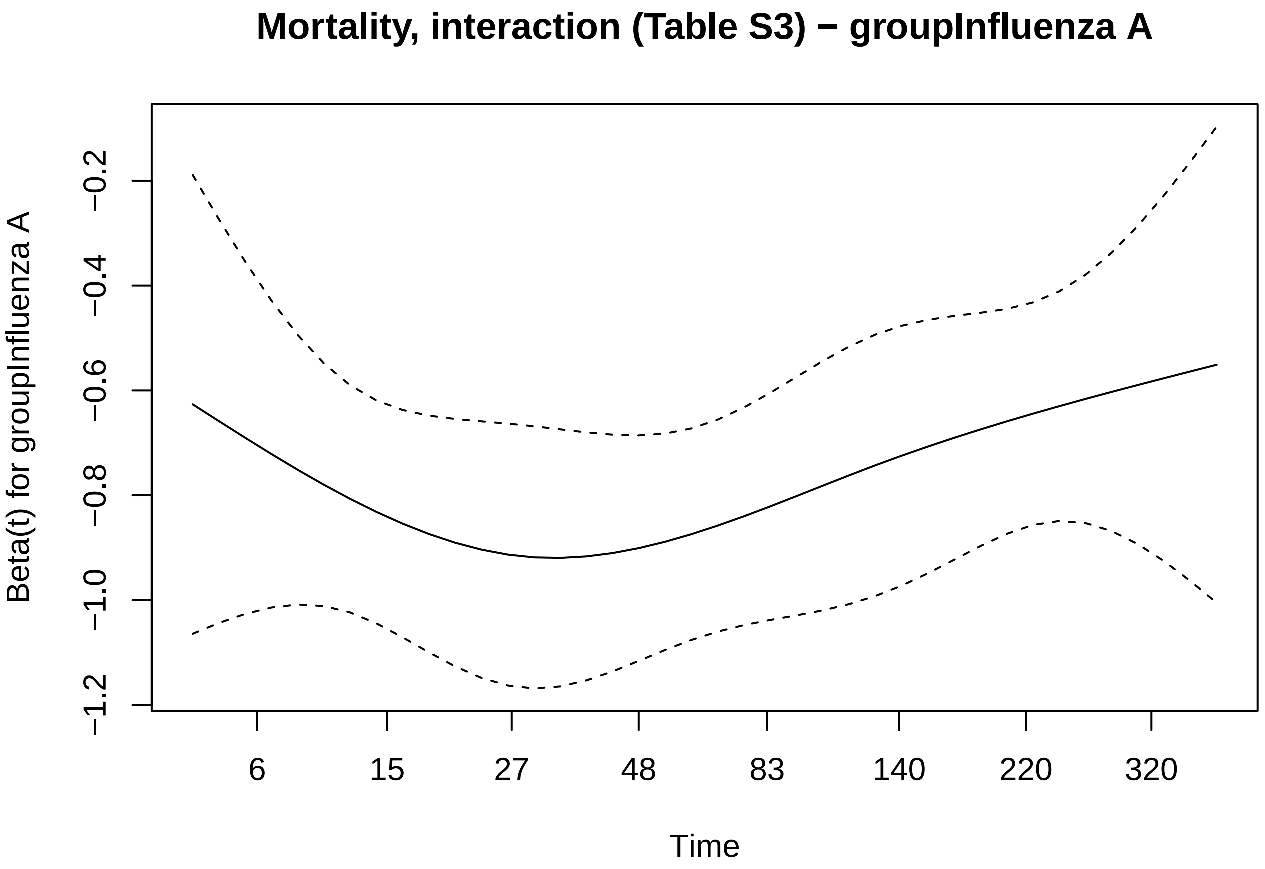
Mortality, interaction (Table S3) − groupInfluenza A
Beta(t) for groupInfluenza A
−0.2
−0.4
−0.6
−0.8
−1.0
−1.2
6
15
27
48
83
140
220
320
Time

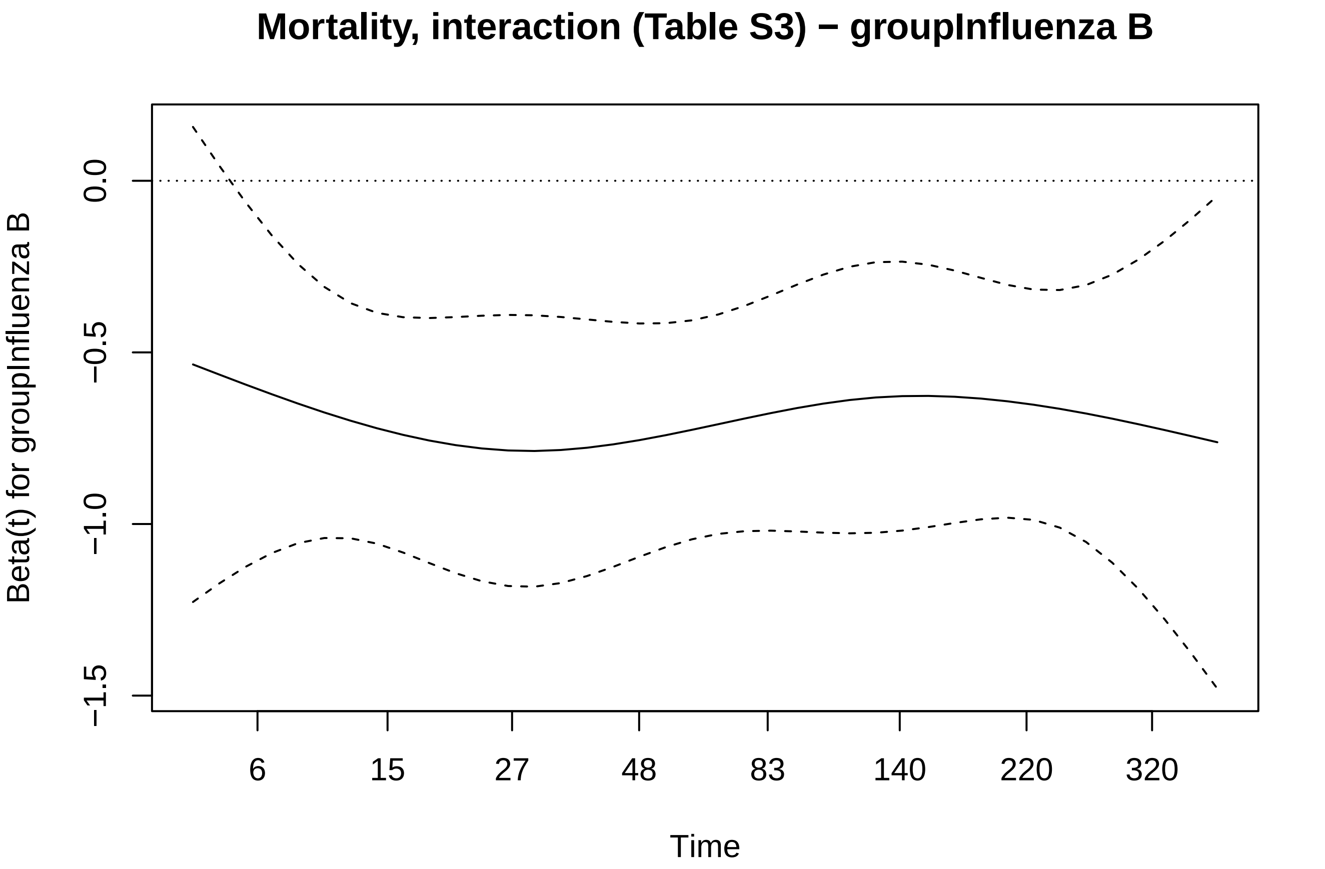
Mortality, interaction (Table S3) − groupInfluenza B
Beta(t) for groupInfluenza B
0.0
−0.5
−1.0
−1.5
6
15
27
48
83
140
220
320
Time

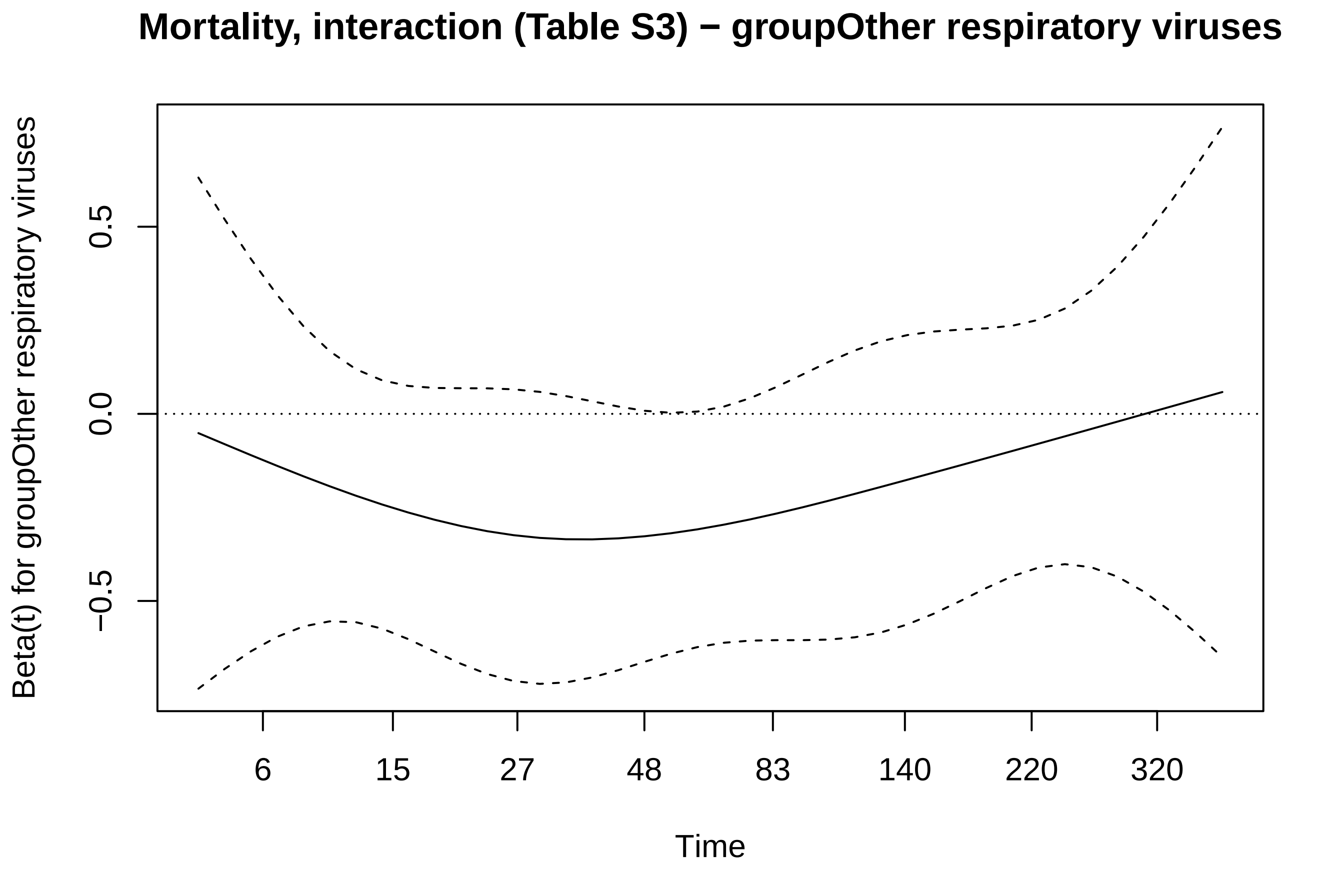
Mortality, interaction (Table S3) − groupOther respiratory viruses
Beta(t) for groupOther respiratory viruses
0.5
0.0
−0.5
6
15
27
48
83
140
220
320
Time

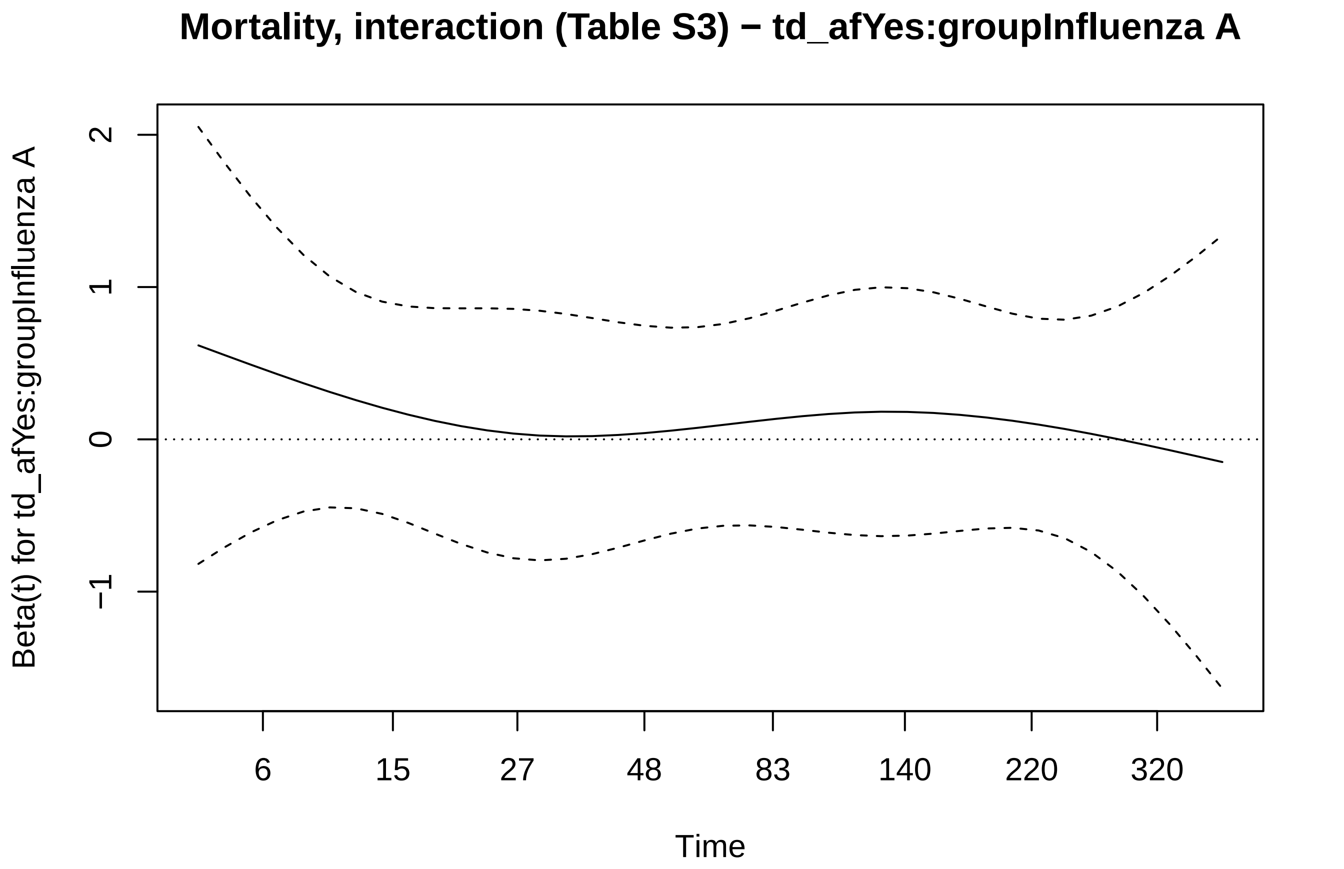
Mortality, interaction (Table S3) − td_afYes:groupInfluenza A
Beta(t) for td_afYes:groupInfluenza A
2
1
0
−1
6
15
27
48
83
140
220
320
Time

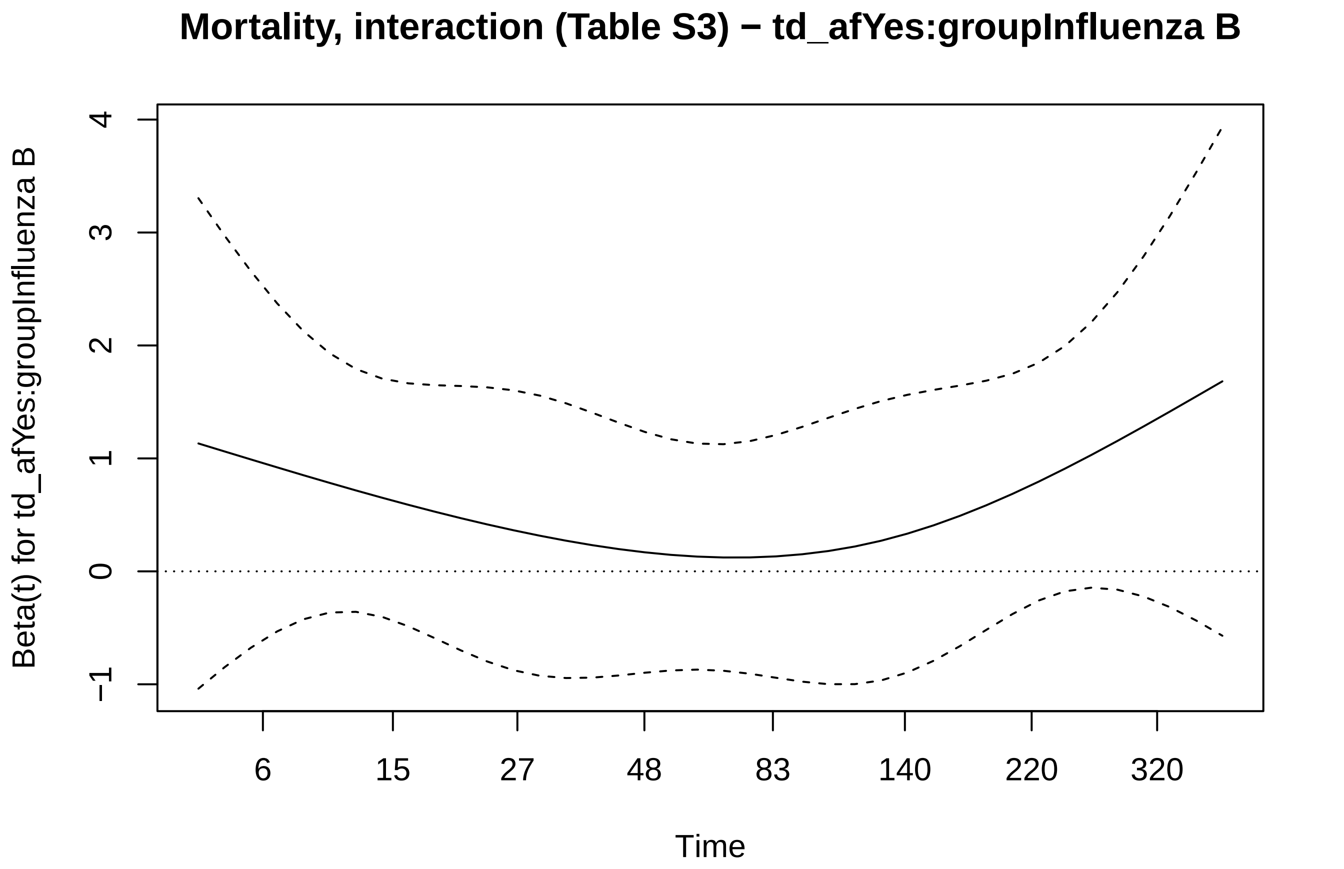
Mortality, interaction (Table S3) − td_afYes:groupInfluenza B
Beta(t) for td_afYes:groupInfluenza B
−1
0
1
2
3
4
6
15
27
48
83
140
220
320
Time

Mortality, interaction (Table S3) − td_afYes:groupOther respiratory viruse

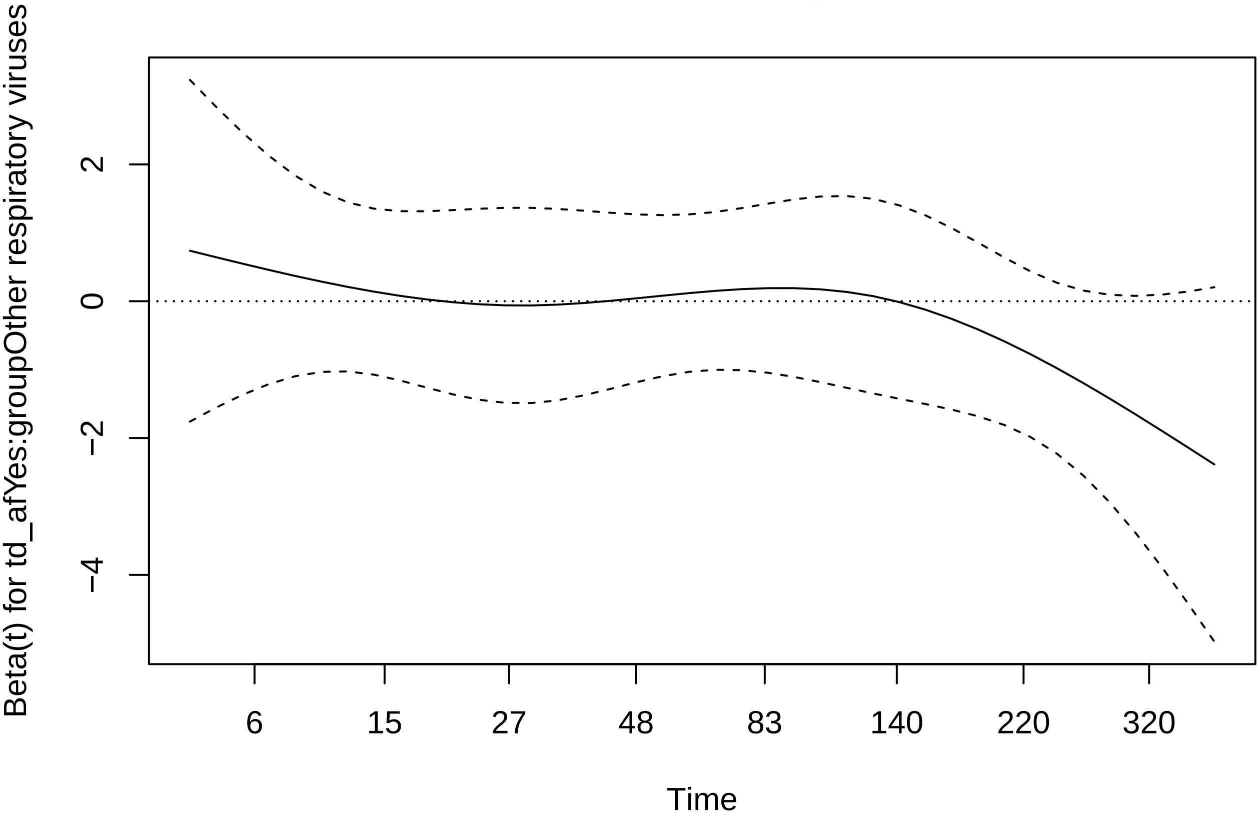

Supplement: Supplementary file 1 [file jcm-15-08608-s001.zip › PH_Schoenfeld_plots.pdf]
