# Supplementary material for: Influenza Infection, Newly Diagnosed Atrial Fibrillation, and One-Year Mortality in Hospitalized Adults: A Retrospective Cohort Study
Source: J Clin Med. 2026 Nov 5;15(21):8608. doi: 10.3390/jcm15218608 (PMC13763677; doi:10.3390/jcm15218608)
Supplement: Supplementary file 1 [file jcm-15-08608-s001.zip › Table_S10_Time_Windows.pdf]

Table S10. NDAF by viral etiology within time windows after admission: numbers at risk, events, and hazard ratios.

Time window	Viral etiology	At risk, n	NDAF events, n	HR (95% CI)
Days 0–30	No virus detected	17,271	683	1 (reference)
	Influenza A	2,829	93	0.89 (0.72–1.11)
	Influenza B	992	23	0.63 (0.42–0.96)
	Other respiratory viruses	561	22	0.86 (0.56–1.32)
Days 31–90	No virus detected	15,451	98	1 (reference)
	Influenza A	2,654	19	1.16 (0.71–1.91)
	Influenza B	938	1	0.17 (0.02–1.22)
	Other respiratory viruses	505	3	0.78 (0.25–2.48)
Days 91–365	No virus detected	14,553	231	1 (reference)
	Influenza A	2,581	28	0.69 (0.46–1.02)
	Influenza B	912	17	1.21 (0.74–1.98)
	Other respiratory viruses	480	10	1.05 (0.55–1.98)
During the index admission	No virus detected	17,205	607	1 (reference)
	Influenza A	2,810	82	0.90 (0.71–1.13)
	Influenza B	985	21	0.66 (0.42–1.01)
	Other respiratory viruses	553	22	1.00 (0.66–1.54)
After discharge	No virus detected	15,795	405	1 (reference)
	Influenza A	2,675	58	0.83 (0.63–1.10)
	Influenza B	947	20	0.83 (0.53–1.30)
	Other respiratory viruses	519	13	0.77 (0.44–1.33)

Time window	Viral etiology	At risk, n	NDAF events, n	HR (95% CI)

Cause-specific Cox models adjusted for the covariates in Table S2; reference group: no virus detected.

Each window includes patients who were alive and free of NDAF at its start. HR, hazard ratio; CI, confidence interval.
