# Supplementary material for: Influenza Infection, Newly Diagnosed Atrial Fibrillation, and One-Year Mortality in Hospitalized Adults: A Retrospective Cohort Study
Source: J Clin Med. 2026 Nov 5;15(21):8608. doi: 10.3390/jcm15218608 (PMC13763677; doi:10.3390/jcm15218608)
Supplement: Supplementary file 1 [file jcm-15-08608-s001.zip › Table_S11_PH_Tests.pdf]

Table S11. Proportional-hazards assumption tests (scaled Schoenfeld residuals) for the main models.

Term	NDAF (Table S2)	Mortality (Table 2)	Mortality, in-hospital/post-discharge (Table S6)	Mortality, interaction (Table S3)	Vaccination, matched (Table 3)
Viral etiology	0.412	0.863	0.855	0.875	–
NDAF (time-dependent)	–	0.179	–	0.188	–
In-hospital NDAF	–	–	0.049	–	–
Post-discharge NDAF	–	–	0.278	–	–
NDAF × viral etiology	–	–	–	0.645	–
Influenza vaccination	–	–	–	–	0.949
Age	0.611	0.001	<0.001	0.001	0.865
Sex	0.424	0.212	0.221	0.212	0.313
Population sector	0.047	0.099	0.097	0.099	0.055
Hypertension	0.241	0.630	0.458	0.629	0.647
Coronary artery disease	0.002	0.551	0.670	0.555	0.076
Congestive heart failure	<0.001	0.165	0.220	0.165	<0.001
Diabetes mellitus	<0.001	0.077	0.108	0.077	<0.001
Chronic kidney disease	0.131	0.168	0.223	0.168	0.261
Obesity	0.540	0.547	0.667	0.553	0.428
Smoking status	0.335	0.318	0.366	0.317	0.268
Alcohol abuse	0.270	0.815	0.761	0.809	0.533
Global test	<0.001	0.022	0.024	0.033	0.006

Values are p-values; –, term not in the model.

In a cohort of this size, small departures from proportional hazards are statistically significant.
