# Supplementary material for: Influenza Infection, Newly Diagnosed Atrial Fibrillation, and One-Year Mortality in Hospitalized Adults: A Retrospective Cohort Study
Source: J Clin Med. 2026 Nov 5;15(21):8608. doi: 10.3390/jcm15218608 (PMC13763677; doi:10.3390/jcm15218608)
Supplement: Supplementary file 1 [file jcm-15-08608-s001.zip › Table_S12_NDAF_Mortality_by_Etiology.pdf]

Table S12. Association between NDAF and one-year mortality within each viral etiology, derived from the interaction model, with numbers of patients, NDAF events, and deaths.

Viral etiology	Patients, n	NDAF, n	Deaths, n	Deaths after NDAF, n	HR for NDAF (95% CI)	Interaction p (Holm-adjusted p)
No virus detected	17,271	1,012	3,589	339	1.66 (1.48–1.85)	Reference
Influenza A	2,829	140	280	26	1.91 (1.27–2.86)	0.508 (1.000)
Influenza B	992	41	108	11	3.01 (1.61–5.61)	0.065 (0.196)
Other respiratory viruses	561	35	108	8	1.34 (0.65–2.75)	0.570 (1.000)

HR for NDAF: hazard of death with versus without NDAF within each viral etiology (time-dependent model, Table S3).

Interaction p: test of whether this HR differs from that in patients with no virus detected; Holm-adjusted for three comparisons.
