# Supplementary material for: Influenza Infection, Newly Diagnosed Atrial Fibrillation, and One-Year Mortality in Hospitalized Adults: A Retrospective Cohort Study
Source: J Clin Med. 2026 Nov 5;15(21):8608. doi: 10.3390/jcm15218608 (PMC13763677; doi:10.3390/jcm15218608)
Supplement: Supplementary file 1 [file jcm-15-08608-s001.zip › Table_S13_Sensitivity.pdf]

Table S13. Sensitivity analyses for NDAF by viral etiology (Part A) and for influenza vaccination and NDAF (Part B). Part A: NDAF by viral etiology (reference: no virus detected).

Analysis	Influenza A, HR (95% CI)	Influenza B, HR (95% CI)	Patients, n	NDAF events, n
Main analysis (Table S2)	0.87 (0.73–1.04)	0.73 (0.53–1.00)	21,653	1,228
Excluding NDAF recorded on the day of admission	0.88 (0.68–1.14)	0.89 (0.59–1.36)	20,968	543
Excluding NDAF recorded on the day of admission (Fine–Gray model)	0.97 (0.75–1.26)	0.99 (0.65–1.49)	20,968	543
Excluding NDAF recorded on days 0–1	0.86 (0.65–1.12)	0.89 (0.58–1.37)	20,945	520
Adjusted for calendar year	0.87 (0.73–1.04)	0.74 (0.54–1.01)	21,653	1,228
Adjusted for calendar year and season	0.84 (0.71–1.01)	0.72 (0.52–0.99)	21,653	1,228
Admissions in December–March only	0.86 (0.71–1.03)	0.75 (0.54–1.03)	16,169	964
Reference group: other respiratory viruses	0.97 (0.67–1.41)	0.81 (0.52–1.28)	21,653	1,228
Known smoking status only (complete-case analysis)	0.88 (0.73–1.08)	0.68 (0.48–0.98)	19,084	988
PCR result within 5 days of admission	0.89 (0.74–1.07)	0.77 (0.56–1.07)	19,829	1,060
Adjusted for PCR ordered from a non-cardiac ICU	0.89 (0.75–1.07)	0.75 (0.55–1.03)	21,653	1,228
Excluding patients tested in any ICU	0.90 (0.75–1.08)	0.75 (0.55–1.04)	20,533	1,104
Including Meir and Yoseftal Medical Centers (original cohort)	0.89 (0.75–1.05)	0.77 (0.59–1.00)	28,562	1,659

In the analysis with other respiratory viruses as the reference group, the HR for no virus detected was 1.12 (0.80–1.56).

HR, hazard ratio; CI, confidence interval; ICU, intensive care unit.

Part B: Influenza vaccination and NDAF (propensity score–matched analyses).

Analysis	Matched pairs, n	NDAF events, vaccinated / unvaccinated	HR (95% CI)
Primary analysis (Table 3)	7,414	485 / 490	0.97 (0.85–1.10)
Calendar year added to the propensity score	7,328	482 / 480	0.99 (0.87–1.13)
Calendar year and season added to the propensity score	6,551	406 / 406	0.98 (0.86–1.13)
Excluding vaccination fewer than 14 days before admission	7,208	492 / 486	0.99 (0.88–1.13)
Viral etiology in both models (original specification)	7,396	507 / 480	1.04 (0.92–1.18)

Robust standard errors clustered on matched pairs.
