# Supplementary material for: Influenza Infection, Newly Diagnosed Atrial Fibrillation, and One-Year Mortality in Hospitalized Adults: A Retrospective Cohort Study
Source: J Clin Med. 2026 Nov 5;15(21):8608. doi: 10.3390/jcm15218608 (PMC13763677; doi:10.3390/jcm15218608)
Supplement: Supplementary file 1 [file jcm-15-08608-s001.zip › Table_S1_Incidence_and_Mortality.pdf]

Table S1. Crude one-year incidence of NDAF by viral etiology and crude one-year mortality by NDAF status.

	NDAF by viral etiology					Mortality by NDAF status	
**Outcome**[1]	**No virus detected**, N = 17,271[2]	**Influenza A**, N = 2,829[2]	**Influenza B**, N = 992[2]	**Other respiratory viruses**, N = 561[2]	**p-value**[3]	**No NDAF**, N = 20,425[2]	**NDAF**, N = 1,228[2]
NDAF within one year	1,012 (5.9%)	140 (4.9%)	41 (4.1%)	35 (6.2%)	0.032		
Death within one year						3,701 (18%)	384 (31%)

[1]Crude proportions. The mortality comparison does not account for when NDAF occurred and is not tested; see Table 2 and Figure 3.

[2]n (%)

[3]Fisher's exact test with simulated p-values (10,000 replicates)
