# Supplementary material for: Influenza Infection, Newly Diagnosed Atrial Fibrillation, and One-Year Mortality in Hospitalized Adults: A Retrospective Cohort Study
Source: J Clin Med. 2026 Nov 5;15(21):8608. doi: 10.3390/jcm15218608 (PMC13763677; doi:10.3390/jcm15218608)
Supplement: Supplementary file 1 [file jcm-15-08608-s001.zip › Table_S2_Cox_NDAF_by_Virus.pdf]

Table S2. Multivariable cause-specific Cox regression for NDAF by viral etiology.

**Characteristic**[1]	**HR**	**95% CI**	**p-value**
Viral etiology			
No virus detected	—	—	
Influenza A	0.87	0.73–1.04	0.118
Influenza B	0.73	0.53–1.00	0.047
Other respiratory viruses	0.90	0.64–1.26	0.524
Age, years	1.04	1.04–1.05	<0.001
Sex			
Female	—	—	
Male	1.14	1.01–1.29	0.032
Population sector			
Jews	—	—	
Arabs	0.85	0.72–1.02	0.077
Other	0.93	0.78–1.12	0.470
Hypertension	1.41	1.19–1.67	<0.001
Coronary artery disease	1.12	0.99–1.28	0.073
Congestive heart failure	1.17	1.01–1.35	0.042
Diabetes mellitus	1.01	0.89–1.14	0.881
Chronic kidney disease	1.11	0.86–1.43	0.430
Obesity	1.38	1.23–1.56	<0.001
Smoking status			
Never	—	—	
Former	0.95	0.81–1.12	0.576
Current	1.12	0.94–1.33	0.204
Unknown	1.00	0.85–1.16	0.975
Alcohol abuse	0.81	0.54–1.23	0.324

[1]n = 21,653; 1,228 NDAF events. Death was treated as a censoring event. HR, hazard ratio; CI, confidence interval.
