# Supplementary material for: Influenza Infection, Newly Diagnosed Atrial Fibrillation, and One-Year Mortality in Hospitalized Adults: A Retrospective Cohort Study
Source: J Clin Med. 2026 Nov 5;15(21):8608. doi: 10.3390/jcm15218608 (PMC13763677; doi:10.3390/jcm15218608)
Supplement: Supplementary file 1 [file jcm-15-08608-s001.zip › Table_S3_Interaction_Mortality.pdf]

Table S3. Time-dependent Cox model with an NDAF × viral etiology interaction for one-year mortality.

**Characteristic**[1]	**HR**	**95% CI**	**p-value**
NDAF (time-dependent)			
No	—	—	
Yes	1.66	1.48–1.85	<0.001
Viral etiology			
No virus detected	—	—	
Influenza A	0.47	0.41–0.53	<0.001
Influenza B	0.50	0.41–0.61	<0.001
Other respiratory viruses	0.83	0.68–1.01	0.061
Age, years	1.04	1.04–1.04	<0.001
Sex			
Female	—	—	
Male	1.24	1.16–1.33	<0.001
Population sector			
Jews	—	—	
Arabs	0.87	0.79–0.95	0.003
Other	1.02	0.92–1.13	0.675
Hypertension	0.96	0.88–1.04	0.309
Coronary artery disease	0.91	0.85–0.99	0.018
Congestive heart failure	1.37	1.26–1.49	<0.001
Diabetes mellitus	1.28	1.20–1.37	<0.001
Chronic kidney disease	1.30	1.13–1.49	<0.001
Obesity	0.82	0.76–0.87	<0.001
Smoking status			
Never	—	—	
Former	1.07	0.98–1.17	0.129
Current	1.11	1.01–1.22	0.023
Unknown	0.99	0.90–1.08	0.772

Characteristic[1]	HR	95% CI	p-value
Alcohol abuse	1.49	1.27–1.75	<0.001
NDAF × viral etiology			
NDAF × Influenza A	1.15	0.76–1.75	0.508
NDAF × Influenza B	1.82	0.96–3.42	0.065
NDAF × Other respiratory viruses	0.81	0.39–1.68	0.570

[1]n = 21,653; 4,085 deaths. Interaction terms show how the HR for NDAF in each virus group differs from that in patients with no virus detected; HRs within each group and Holm-adjusted p-values are in Table S12. HR, hazard ratio; CI, confidence interval.
