# Supplementary material for: Influenza Infection, Newly Diagnosed Atrial Fibrillation, and One-Year Mortality in Hospitalized Adults: A Retrospective Cohort Study
Source: J Clin Med. 2026 Nov 5;15(21):8608. doi: 10.3390/jcm15218608 (PMC13763677; doi:10.3390/jcm15218608)
Supplement: Supplementary file 1 [file jcm-15-08608-s001.zip › Table_S4_Cox_Vaccination_Unmatched.pdf]

Table S4. Cox models for influenza vaccination and NDAF in the full (unmatched) cohort.

	Unadjusted			Adjusted, with viral etiology			Adjusted, without viral etiology		
**Characteristic[1]**	**HR**	**95% CI**	**p-value**	**HR**	**95% CI**	**p-value**	**HR**	**95% CI**	**p-value**
Influenza vaccination within 6 months	1.68	1.50–1.88	<0.001	1.00	0.89–1.12	0.935	0.99	0.88–1.12	0.897
Viral etiology									
No virus detected				—	—				
Influenza A				0.87	0.73–1.04	0.119			
Influenza B				0.73	0.53–1.00	0.047			
Other respiratory viruses				0.90	0.64–1.26	0.526			
Age, years				1.04	1.04–1.05	<0.001	1.04	1.04–1.05	<0.001
Sex									
Female				—	—		—	—	
Male				1.14	1.01–1.29	0.032	1.14	1.01–1.29	0.031
Population sector									
Jews				—	—		—	—	
Arabs				0.86	0.72–1.02	0.077	0.86	0.72–1.02	0.083
Other				0.93	0.78–1.12	0.467	0.94	0.78–1.13	0.498
Hypertension				1.41	1.19–1.67	<0.001	1.41	1.19–1.67	<0.001

	Unadjusted			Adjusted, with viral etiology			Adjusted, without viral etiology		
**Characteristic**[1]	**HR**	**95% CI**	**p-value**	**HR**	**95% CI**	**p-value**	**HR**	**95% CI**	**p-value**
Coronary artery disease				1.12	0.99–1.28	0.073	1.12	0.99–1.28	0.074
Congestive heart failure				1.17	1.01–1.35	0.042	1.17	1.01–1.36	0.036
Diabetes mellitus				1.01	0.89–1.14	0.878	1.01	0.89–1.14	0.864
Chronic kidney disease				1.11	0.86–1.43	0.430	1.11	0.86–1.43	0.420
Obesity				1.38	1.23–1.56	<0.001	1.38	1.23–1.56	<0.001
Smoking status									
Never				—	—		—	—	
Former				0.95	0.81–1.12	0.577	0.96	0.81–1.13	0.593
Current				1.12	0.94–1.33	0.205	1.12	0.94–1.33	0.191
Unknown				1.00	0.86–1.16	0.976	1.00	0.86–1.16	0.982
Alcohol abuse				0.81	0.54–1.23	0.324	0.82	0.54–1.24	0.342

[1]n = 21,653; 1,228 NDAF events. HR, hazard ratio; CI, confidence interval.
