# Supplementary material for: Influenza Infection, Newly Diagnosed Atrial Fibrillation, and One-Year Mortality in Hospitalized Adults: A Retrospective Cohort Study
Source: J Clin Med. 2026 Nov 5;15(21):8608. doi: 10.3390/jcm15218608 (PMC13763677; doi:10.3390/jcm15218608)
Supplement: Supplementary file 1 [file jcm-15-08608-s001.zip › Table_S5_Fine_Gray.pdf]

Table S5. Fine–Gray competing-risk regression for NDAF, with death as the competing event.

**Characteristic**[1]	**sHR**	**95% CI**	**p-value**
Viral etiology			
No virus detected	—	—	
Influenza A	0.91	0.76–1.08	0.290
Influenza B	0.76	0.56–1.04	0.082
Other respiratory viruses	0.91	0.65–1.28	0.605
Age, years	1.04	1.04–1.05	<0.001
Sex			
Female	—	—	
Male	1.13	1.00–1.27	0.045
Population sector			
Jews	—	—	
Arabs	0.86	0.72–1.03	0.098
Other	0.94	0.78–1.13	0.491
Hypertension	1.42	1.19–1.70	<0.001
Coronary artery disease	1.13	0.99–1.29	0.067
Congestive heart failure	1.14	0.98–1.32	0.093
Diabetes mellitus	1.00	0.88–1.13	0.996
Chronic kidney disease	1.08	0.84–1.40	0.543
Obesity	1.41	1.25–1.58	<0.001
Smoking status			
Never	—	—	
Former	0.95	0.81–1.12	0.554
Current	1.11	0.94–1.32	0.227
Unknown	1.00	0.85–1.17	0.975
Alcohol abuse	0.79	0.52–1.19	0.262

[1]n = 21,653; 1,228 NDAF events. sHR, subdistribution hazard ratio; CI, confidence interval.
