# Supplementary material for: Influenza Infection, Newly Diagnosed Atrial Fibrillation, and One-Year Mortality in Hospitalized Adults: A Retrospective Cohort Study
Source: J Clin Med. 2026 Nov 5;15(21):8608. doi: 10.3390/jcm15218608 (PMC13763677; doi:10.3390/jcm15218608)
Supplement: Supplementary file 1 [file jcm-15-08608-s001.zip › Table_S6_InHospital_PostDischarge_Mortality.pdf]

Table S6. Time-dependent Cox model with separate indicators for in-hospital and post-discharge NDAF and one-year mortality.

**Characteristic**[1]	**HR**	**95% CI**	**p-value**
In-hospital NDAF (time-dependent)	1.32	1.16–1.51	<0.001
Post-discharge NDAF (time-dependent)	3.04	2.57–3.59	<0.001
Viral etiology			
No virus detected	—	—	
Influenza A	0.47	0.42–0.53	<0.001
Influenza B	0.53	0.43–0.64	<0.001
Other respiratory viruses	0.82	0.67–0.99	0.037
Age, years	1.04	1.04–1.04	<0.001
Sex			
Female	—	—	
Male	1.24	1.16–1.32	<0.001
Population sector			
Jews	—	—	
Arabs	0.86	0.79–0.95	0.002
Other	1.02	0.92–1.13	0.663
Hypertension	0.96	0.88–1.04	0.347
Coronary artery disease	0.92	0.85–0.99	0.021
Congestive heart failure	1.37	1.26–1.49	<0.001
Diabetes mellitus	1.27	1.19–1.37	<0.001
Chronic kidney disease	1.27	1.11–1.46	<0.001
Obesity	0.82	0.76–0.87	<0.001
Smoking status			
Never	—	—	
Former	1.07	0.98–1.17	0.114
Current	1.11	1.02–1.22	0.020
Unknown	1.00	0.91–1.09	0.952
Alcohol abuse	1.49	1.27–1.75	<0.001

Characteristic[1]	HR	95% CI	p-value

[1]In-hospital NDAF: 732 patients (236 deaths after NDAF); post-discharge NDAF: 496 patients (148 deaths after NDAF). HR, hazard ratio; CI, confidence interval.
