# Supplementary material for: Influenza Infection, Newly Diagnosed Atrial Fibrillation, and One-Year Mortality in Hospitalized Adults: A Retrospective Cohort Study
Source: J Clin Med. 2026 Nov 5;15(21):8608. doi: 10.3390/jcm15218608 (PMC13763677; doi:10.3390/jcm15218608)
Supplement: Supplementary file 1 [file jcm-15-08608-s001.zip › Table_S7_Baseline_by_NDAF.pdf]

Table S7. Baseline characteristics by NDAF status.

Characteristic	**No NDAF**, N = 20,425[1]	**NDAF**, N = 1,228[1]	**SMD**[2]
**Viral etiology**			0.09
No virus detected	16,259 (80%)	1,012 (82%)	
Influenza A	2,689 (13%)	140 (11%)	
Influenza B	951 (4.7%)	41 (3.3%)	
Other respiratory viruses	526 (2.6%)	35 (2.9%)	
**Age, years**	62 ± 21	76 ± 12	0.89
**Sex**			0.05
Female	10,688 (52%)	614 (50%)	
Male	9,737 (48%)	614 (50%)	
**Population sector**			0.24
Jews	14,420 (71%)	937 (76%)	
Arabs	4,395 (22%)	157 (13%)	
Other	1,610 (7.9%)	134 (11%)	
**Hypertension**	11,216 (55%)	1,010 (82%)	0.62
**Coronary artery disease**	4,712 (23%)	483 (39%)	0.36
**Congestive heart failure**	2,395 (12%)	268 (22%)	0.27

**Characteristic**	**No NDAF**, N = 20,425[1]	**NDAF**, N = 1,228[1]	**SMD**[2]
**Diabetes mellitus**	5,560 (27%)	481 (39%)	0.26
**Chronic kidney disease**	786 (3.8%)	65 (5.3%)	0.07
**Obesity**	7,870 (39%)	627 (51%)	0.25
**Smoking status**			0.24
Never	10,987 (54%)	598 (49%)	
Former	3,256 (16%)	206 (17%)	
Current	3,853 (19%)	184 (15%)	
Unknown	2,329 (11%)	240 (20%)	
**Alcohol abuse**	573 (2.8%)	24 (2.0%)	0.06
**Influenza vaccination within 6 months**	8,539 (42%)	670 (55%)	0.26
**PCR ordered from an intensive care unit**	996 (4.9%)	124 (10%)	0.20

[1]n (%); mean ± SD

[2]Absolute standardized mean difference between patients with and without NDAF.
