# Supplementary material for: Influenza Infection, Newly Diagnosed Atrial Fibrillation, and One-Year Mortality in Hospitalized Adults: A Retrospective Cohort Study
Source: J Clin Med. 2026 Nov 5;15(21):8608. doi: 10.3390/jcm15218608 (PMC13763677; doi:10.3390/jcm15218608)
Supplement: Supplementary file 1 [file jcm-15-08608-s001.zip › Table_S8_Vaccination_Before_After_Matching.pdf]

Table S8. Baseline characteristics of vaccinated and unvaccinated patients before and after propensity score matching, with standardized mean differences.

	Before matching			After matching		
**Characteristic**	**Not vaccinated**, N = 12,444[1]	**Vaccinated**, N = 9,209[1]	**SMD**[2]	**Not vaccinated**, N = 7,414[1]	**Vaccinated**, N = 7,414[1]	**SMD**[2]
**Viral etiology**			0.09			0.08
No virus detected	9,969 (80%)	7,302 (79%)		6,002 (81%)	5,864 (79%)	
Influenza A	1,640 (13%)	1,189 (13%)		922 (12%)	968 (13%)	
Influenza B	586 (4.7%)	406 (4.4%)		322 (4.3%)	325 (4.4%)	
Other respiratory viruses	249 (2.0%)	312 (3.4%)		168 (2.3%)	257 (3.5%)	
**Age, years**	57 ± 21	70 ± 17	0.71	68 ± 17	68 ± 17	0.01
**Sex**			0.07			0.01
Female	6,693 (54%)	4,609 (50%)		3,842 (52%)	3,810 (51%)	
Male	5,751 (46%)	4,600 (50%)		3,572 (48%)	3,604 (49%)	
**Population sector**			0.14			0.04
Jews	8,526 (69%)	6,831 (74%)		5,368 (72%)	5,401 (73%)	
Arabs	2,770 (22%)	1,782 (19%)		1,387 (19%)	1,432 (19%)	
Other	1,148 (9.2%)	596 (6.5%)		659 (8.9%)	581 (7.8%)	
**Hypertension**	5,588 (45%)	6,638 (72%)	0.57	4,932 (67%)	4,937 (67%)	0.00

	Before matching			After matching		
**Characteristic**	**Not vaccinated**, N = 12,444[1]	**Vaccinated**, N = 9,209[1]	**SMD**[2]	**Not vaccinated**, N = 7,414[1]	**Vaccinated**, N = 7,414[1]	**SMD**[2]
**Coronary artery disease**	2,185 (18%)	3,010 (33%)	0.35	2,026 (27%)	2,110 (28%)	0.03
**Congestive heart failure**	1,045 (8.4%)	1,618 (18%)	0.28	976 (13%)	1,070 (14%)	0.04
**Diabetes mellitus**	2,550 (20%)	3,491 (38%)	0.39	2,342 (32%)	2,439 (33%)	0.03
**Chronic kidney disease**	374 (3.0%)	477 (5.2%)	0.11	320 (4.3%)	358 (4.8%)	0.02
**Obesity**	4,457 (36%)	4,040 (44%)	0.17	3,136 (42%)	3,164 (43%)	0.01
**Smoking status**			0.29			0.02
Never	6,928 (56%)	4,657 (51%)		3,850 (52%)	3,845 (52%)	
Former	1,719 (14%)	1,743 (19%)		1,319 (18%)	1,306 (18%)	
Current	2,669 (21%)	1,368 (15%)		1,277 (17%)	1,250 (17%)	
Unknown	1,128 (9.1%)	1,441 (16%)		968 (13%)	1,013 (14%)	
**Alcohol abuse**	369 (3.0%)	228 (2.5%)	0.03	192 (2.6%)	202 (2.7%)	0.01

[1]n (%); mean ± SD

[2]Absolute standardized mean difference between vaccinated and unvaccinated patients. Viral etiology was not used for matching.
