# Supplementary material for: Influenza Infection, Newly Diagnosed Atrial Fibrillation, and One-Year Mortality in Hospitalized Adults: A Retrospective Cohort Study
Source: J Clin Med. 2026 Nov 5;15(21):8608. doi: 10.3390/jcm15218608 (PMC13763677; doi:10.3390/jcm15218608)
Supplement: Supplementary file 1 [file jcm-15-08608-s001.zip › Table_S9_By_Year.pdf]

Table S9. Number of tested patients, viral etiology, and influenza vaccination by calendar year, 2012–2020.

Year	Patients, n	No virus detected, %	Influenza A, %	Influenza B, %	Other respiratory viruses, %	Vaccinated, %
2012	191	85.3	10.5	3.1	1.0	29.8
2013	673	72.8	23.8	1.3	2.1	37.6
2014	1,126	82.4	12.1	3.6	2.0	41.4
2015	2,079	82.5	12.3	2.6	2.5	40.3
2016	3,128	80.7	11.1	6.2	2.0	47.8
2017	3,321	81.2	12.0	5.6	1.3	45.1
2018	4,113	80.0	9.1	8.7	2.2	42.7
2019	4,960	79.4	16.5	0.4	3.7	37.9
2020	2,062	74.0	15.8	6.0	4.3	47.0
Total	21,653	79.8	13.1	4.6	2.6	42.5

2020 includes January and February only.
